# Evaluation of the relationship between occupational-specific task performance and measures of physical fitness, cardiovascular and musculoskeletal health in firefighters

**DOI:** 10.1186/s12889-023-17487-6

**Published:** 2024-01-02

**Authors:** Jaron Ras, Elpidoforos S. Soteriades, Denise L. Smith, Andre P. Kengne, Lloyd Leach

**Affiliations:** 1 Department of Sport, Recreation and Exercise Science, Faculty of Community and Health Sciences, University of the Western Cape, Cape Town, South Africa; 2 Healthcare Management Program, School of Economics and Management, Open University of Cyprus, Nicosia, Cyprus; 3 Environmental and Occupational Medicine and Epidemiology (EOME), Department of Environmental Health, Harvard T.H. Chan School of Public Health, Boston, MA USA; 4 Health and Human Physiological Sciences, Skidmore College, Saratoga Springs, New York USA; 5 Non-Communicable Diseases Research Unit, South African Medical Research Council, Cape Town, South Africa

**Keywords:** Firefighting, Task performance, Physical fitness, Obesity, Hypertension, Cardiorespiratory fitness, Strength, Endurance, Injury, Discomfort

## Abstract

**Introduction:**

Firefighters are required to perform physically strenuous tasks such as hose drags, victim rescues, forcible entries and stair climbs to complete their public safety mission. Occupational-specific tasks are often used to evaluate the ability of firefighters to adequately/safely perform their duties. Depending on the regions, occupational-specific tasks include six to eight individual tasks, which emphasize distinct aspects of their physical fitness, while also requiring different levels of cardiovascular (CVH) and musculoskeletal health (MSH). Therefore, the aim of this study was to evaluate the relationship between specific occupational task performance and measures of physical fitness, cardiovascular and musculoskeletal health.

**Methods:**

Using a cross-sectional design, 282 full-time male and female firefighters were recruited. A researcher-generated questionnaire and physical measures were used to collect data on sociodemographic characteristics, CVH, MSH and weekly physical activity habits. Physical measures were used to collect data on physical fitness and occupational-specific task performance.

**Results:**

Absolute cardiorespiratory fitness (abV̇O2max), grip strength, leg strength, push-ups, sit-ups and lean body mass (all *p* < 0.001) had an inverse association with completion times on all occupational-specific tasks. Age was positively related to the performance of all tasks (all *p* < 0.05). Higher heart rate variability (HRV) was associated with better performance on all tasks (all *p* < 0.05). Bodyfat percentage (BF%) and diastolic blood pressure were positively associated with the step-up task (*p* < 0.05). Lower back musculoskeletal injury (LoBMSI), musculoskeletal discomfort (MSD), and lower limb MSD were associated with a decreased odds of passing the step-up. Upper body MSIs (UBMSI), LoBMSIs and Lower back MSD were associated with decreased odds of passing the rescue drag.

**Conclusion:**

Firefighters that were taller, leaner, stronger and fitter with a more favourable CVH profile, higher HRV and less musculoskeletal discomfort performed best on all occupational-specific tasks.

**Supplementary Information:**

The online version contains supplementary material available at 10.1186/s12889-023-17487-6.

## Introduction

Firefighting is a strenuous and challenging occupation where firefighters are required to be prepared, at all times, to respond to fire and rescue emergencies. Some of these emergencies, especially those on the fire ground, require high levels of physical exertion, which often entail coping with environmental stressors, such as high temperatures, physical hazards and dangerous chemicals and fumes, [1–3]. The harsh environments often require firefighters to be encapsulated in personal protective equipment (PPE), placing an additional burden on an already strained cardiovascular and musculoskeletal system [3–5]. The strenuous work conditions of firefighting necessitate that firefighters maintain peak physical conditioning to manage these various and, often, unpredictable high-demand environments and situations [5–7].

Although firefighting elicits near maximum physiological responses, placing significant strain on the cardiovascular system, studies have found that firefighters often have multiple cardiovascular disease (CVD) risk factors and poor overall cardiovascular health (CVH) [8–10]. The cardiovascular risk profile of firefighters progressively worsens as they age [11, 12]. In addition, despite many firefighters possessing the ability to perform the necessary work-related tasks required in firefighting, many firefighters are reported to not meet the minimum physical fitness levels required for the profession [3, 13–16], placing an additional burden on an already strained cardiovascular and musculoskeletal system [1–3, 5, 6]. Low levels of CVH and physical fitness are prominent precursors contributing to the high incidence of cardiac events and over-exertion related incidents, which account for 40 to 50% of all on-duty fatalities among firefighters [1, 2, 4]. To cope with the physiological and psychological stressors of the job firefighters need good cardiovascular and musculoskeletal health (MSH) and acceptable level of physical fitness [3, 5, 6].

Previous research has indicated that age and obesity were associated with significantly reduced occupational performance of firefighters, particularly for duties requiring heavy lifting and dragging [3, 5, 17, 18]. Activities that include a large static component may provide an exaggerated blood pressure response, especially if the tasks require overhead movements, which may be especially prominent in firefighters suffering from blood pressure irregularities [19–21]. Firefighters are encouraged by fire departments to remain physically active to ensure they maintain an adequate level of physical fitness. Previous studies have indicated that cardiorespiratory fitness may be the most important factor contributing to adequate occupational performance [22, 23]. In addition, a higher level of muscle strength and endurance has been shown to improve occupational performance, particularly for tasks involving heavy lifting, dragging, pulling and breaching [3, 5, 6, 18]. An added benefit of firefighters remaining physically active is the preservation of MSH, which constitutes a major concern in the profession [24, 25]. Deterioration of MSH, which is common in firefighters, may reduce occupational performance due to guarding of the painful area [26, 27] or reduced force production as a protective mechanism. Firefighting requires firefighters to perform awkward movement patterns to perform their duties, while carrying asymmetrical loads [27–29]. It has been suggested that previous musculoskeletal injuries (MSIs) or current MSD may impact firefighters’ effectiveness in performing specific body movements [26]. Thus, firefighters are required to maintain high levels of work functioning in all occupational-specific tasks [27, 30, 31].

To assess firefighters’ occupational performance, fire departments use simulation protocols designed to replicate the duties that firefighters are required to perform [5, 6, 32, 33]. Each occupational-specific task reflects a core or critical task that firefighters are required to perform, such as the forcible entry, hose drag, ladder raise and victim rescue [3, 5, 6]. The performance of each task is timed to ensure firefighters are able to complete their duties with sufficient rigour and intensity. In addition, to pass the occupational-specific tasks, firefighters are required to complete each task within a given time limit. Several studies have assessed the relationship between physical fitness [3, 5, 6, 18], specific CVH [3, 18, 34] and MSH [27] parameters and occupational performance in firefighters. However, there remains a need to evaluate the relationship between performance on each of the individual occupational-specific tasks and measures of physical fitness, CVH and MSH, warranting further investigation. Determining the factors influencing specific firefighter task performance in this population may highlight the tasks firefighters are most likely to fail and assist in the establishment of intervention strategies to assist firefighters in improving their performance. Therefore, the aim of this study was to evaluate the performance of occupational-specific tasks in association with firefighters’ physical fitness, CVH and MSH.

## Methods and materials

### Study design and population

A cross-sectional study design was employed to collect information on occupational performance, using occupational-specific tasks (based on the physical ability test), physical fitness (cardiorespiratory fitness, muscular strength and endurance, flexibility, and body composition), CVH (CVD risk factors, CVH metrics, heart rate variability) and MSH (MSIs and MSD) in firefighters. In total, 309 full-time male and female firefighters from the City of Cape Town Fire and Rescue Service (CoCTFRS), ranging in age from 20 to 65 years, took part in the study. From the original 309 firefighters, 283 agreed to participate in the occupational-specific tasks on the day of testing. Amongst the 282 that performed the occupational-specific tasks, 268 completed all occupational-specific tasks that were part of the PAT. However, 18.7% failed to complete the occupational-specific task battery in the required time or failed to complete all tasks. In addition, three firefighters failed to complete the first task (step-up). All volunteers for this study provided written informed consent before proceeding. Data collection took place from June to August of 2022. The University of the Western Cape's Biomedical Research Ethics Committee gave its approval (ethical clearance number: BM21/10/9). The Chief Fire Officer, the Department of Policy and Strategy, and the research all gave their approval.

### Sampling and participant recruitment

Data collection took place during annual physical fitness assessments at a standardized fire station located in the City of Cape Town (CCT) metropolitan area to assure consistency in the terrain, environmental conditions and testing surface. To ensure the consistency and reliability of the testing results, all physical measures and the occupational-specific tasks were collected and recorded by trained researchers that were familiarised with all the testing instruments and research procedures [35]. Every third firefighter from the 96 platoons (32 fire stations) was selected using random systematic sampling. The 96 firefighter platoons each had 8 to 12 members. All firefighters that were between the ages of 20–65 years were eligible to participate in the study. Firefighters who were on administrative duty, sick leave, worked part-time or seasonally, or did not participate in the PAT, on the day of testing, were all disqualified from partaking in this study.

### Occupational-specific tasks

The occupational-specific tasks were used to assess operational performance and were conducted according to the testing protocol of the CoCTFRS wellness manual. The CoCTFRS worked with professionals in the field to establish the occupational-specific tasks as part of the fitness and wellness programme. The occupational-specific tasks consisted of tasks that are intended to replicate the numerous tasks that firefighters are required to carry out, while also attempting to replicate the physical strains to which firefighters are frequently exposed to. Firefighters were required to complete the entire simulation protocol in under 9 min (540 s), which included the allowed 20 s of recovery between tasks. Firefighters wore their full PPE equipment and breathing apparatus set, in order to pass. The simulation included six tasks, which were used to simulate various stressors firefighters are placed under. These tasks encompassed the step-up, charged hose drag and pull, forcible entry, equipment carry, ladder raise and extension and the rescue drag. Individual occupational-specific tasks each had their own completion times that needed to be met in order to pass the  testing battery. Failure to complete a task resulted in firefighters being graded as “not yet competent”. The step-up required firefighters to perform 30 step-ups on a standardized platform of 200 mm and were given a time limit of 90 s. The charged hose drag and pull required firefighters to drag a tyre 27 m, drop to one knee or in a seated position, pull a tyre another 15 m and had a time limit of 180 s. The firefighters moved to the forcible entry task where they were required pick up a 6-kg sledgehammer to drive the tyre 600 mm in under 60 s. For the equipment carry, firefighters were tasked to remove two 25 kg foam drums from a 1.2-m platform, carry the foam drums 25 m and walk back another 25 m, placing the drums back on the platform which needed to be completed in under 90 s. For the ladder raise and extension firefighters were tasked to walk a seven-to-eight-meter ladder toward a building, place the ladder against the building and immediately walk toward a hauling line and hoist a 35 kg drum until it reaches the pulley and then lower the drum, in under the time limit of 90 s. Then, firefighters lower the ladder and walk the ladder back to the starting position. The rescue drag required firefighters to grasp an 80 kg tyre and drag the tyre 11 m, perform a 180-degree turn and continue for another 11 m toward the finish line in under 60 s. A full description of the occupational-specific tasks can be found in Ras et al. [35].

### Physical fitness measures

Physical fitness was measured using the American College of Sports Medicine (ACSM) guidelines [36]. Cardiorespiratory capacity was calculated using a validated non-exercise calculation [35, 37] to determine oxygen consumption (V̇O_2_). The push-ups and sit-ups tests were used to assess muscular endurance, handgrip and leg strength tests were used to assess upper and lower body muscle strength and the sit-and-reach test was used to assess flexibility. Body mass and Lean body mass (LBM) was used as a measure for body composition and assessed using a bioelectrical impedance (BIA) analyser (Tanita© BC-1000 Plus BIA scale). For a full description of the methods used to assess physical fitness consult the study published by: Ras et al. [38].

### Classification of physical fitness parameters

For relative cardiorespiratory fitness, 42 mL•kg•min [39] was used to indicate the minimum cardiorespiratory fitness needed for firefighting. Cardiorespiratory fitness was expressed as both absolute and relative cardiorespiratory fitness and odds ratios were calculated on both separately. Due to the absence of standardized minimum requirements of absolute cardiorespiratory fitness, muscular strength, endurance and flexibility, the 50th percentile was used to indicate good levels of physical fitness. Absolute cardiorespiratory fitness was considered the maximum oxygen consumed in one minute and relative cardiorespiratory fitness was considered as the relative oxygen consumed, relative to lean body mass [40–42]. An absolute cardiorespiratory fitness level of 3.40 L•min was considered “good”. For grip and leg strength, firefighters that had a grip strength above 89.9 kg and leg strength above 116.5 kg were considered “good”. For push-ups and sit-ups, firefighters that performed 30 or more push-ups and sit-ups were considered “good”. For flexibility, a sit-and-reach above 43 cm was considered “good”. Firefighters falling below the 50th percentile were classified as having a “low” level of muscular strength and endurance and flexibility.

### Cardiovascular health measures

Cardiovascular health (CVH) was investigated using several approaches. These approaches included three main subcomponents, specifically traditional CVD risk factors, CVH metrics and heart rate variability (HRV). Using standardized techniques [36], height was measured with a stadiometer and waist and hip circumference were assessed using a tape measure, and body fat percentage (BF%) was calculated using a BIA scale. The traditional CVD risk factors included age, obesity, physical inactivity, dyslipidaemia, diabetes, hypertension and cigarette smoking. Cardiovascular health metrics were used to classify firefighters’ cardiovascular health index (CVHI). The CVH metrics included smoking status, blood pressure, non-fasting blood glucose (NFBG), total cholesterol (TC), an ideal/good body mass index (BMI), level of physical activity, and diet. In addition, CVHI was classified as “poor” if firefighters had zero to two metrics classified as ideal, “intermediate” if firefighters had three to four metrics classified as ideal and “good” if firefighters had five to seven metrics rated as ideal. The 2008 Framingham risk model, developed by D'Agostino et al. [43], was used to assess cardiovascular risk of firefighters. The 2008 Framingham risk model, developed by D'Agostino et al. [43], was used to assess cardiovascular disease risk of firefighters. In addition, to determine the cardiovascular disease risk among firefighters, the American College of Cardiology (ACC) 10-year atherosclerotic cardiovascular disease (ASCVD) and ASCVD lifetime risks were calculated [44, 45]. For HRV, a Polar™ (Polar Electro Oy, Kempele, Finland) H10 heart rate monitor was used, at rest, while firefighters were in a seated position, and analyzed using the Kubio© Software version 3.4.3. Moreover, the following HRV measures were collected: standard deviation of all normal-to-normal (SDNN); root-mean-square of successive differences (RMSSD; low-frequency (LF); high frequency (HF); low and high frequency ratio (LF/HF) [46, 47]. For more information on the methods used to assess CVH, as well as the classifications of CVD risk factors and CVH metrics, please refer to Ras et al. [48].

### Classification of musculoskeletal health

Musculoskeletal health was subdivided as musculoskeletal injury (MSI) and musculoskeletal discomfort (MSD) status, which was further separated into those that sustained an injury while on duty and those that did not, and those that are experiencing MSD and those without. Musculoskeletal injury and discomfort were measured subjectively via two validated questionnaires, namely the Cornell Musculoskeletal Discomfort Questionnaire [49] and the Nordic Musculoskeletal Questionnaire. Subcategories for those that reported MSIs and MSD were categorized based on the location of the MSI or the MSD experienced, specifically upper body MSI (UBMSI), lower body MSI (LBMSI), lower back MSI (LoBMSI) upper body MSD (UBMSD), lower body musculoskeletal discomfort (LBMSD) and lower back MSD (LoMSD).

### Statistical analysis

The data were analysed using SPSS® software, version 28 (Chicago, Illinois, USA). Descriptive statistical analyses, such as the median and 25th and 75th percentiles were performed. Thereafter, group comparisons used the Mann–Whitney U and Kruskal–Wallis H test. Univariable and multivariable linear regressions were performed to determine the independent variables associated with occupational-specific tasks, i.e., step-up, charged hose drag and pull, forcible entry, equipment carry, ladder raise and extension and rescue drag, which was considered the outcome (dependent variable) in firefighters. Completion time for each tasks was recorded to nearest second. Univariable and multivariable logistic regressions were performed to determine the independent variables associated with the occupational-specific tasks pass rates. Pass rates were calculated from predetermined cut-off values. Exploratory physical fitness variables included abV̇O2max, relV̇O2max, grip strength, leg strength, push-ups, sit-ups, and LBM. Exploratory CVH variables included age, BMI, BF%, WC, SBP, DBP, TC, NFBG, weekly MET minutes and Framingham risk score. Exploratory variables for MSH included MSI, upper body musculoskeletal injury (UBMSI), lower body musculoskeletal injury (LBMSI), lower back musculoskeletal injury (LoBMSI), MSD, lower back musculoskeletal discomfort (LoBMSD), upper body musculoskeletal discomfort (UBMSD) and lower body musculoskeletal discomfort (LBMSD). Multivariable model 2 were adjusted for age, sex, height and weekly metabolic equivalent minutes. For variables which remained significant, additional multivariable models were run where covariates included physical fitness, CVH and MSH. In addition, to reduce the number of independent variables and likelihood of multicollinearity, principal components analysis (PCA) was run on physical fitness and CVH variables to discern the variables explaining the most variability in physical fitness and CVH. The Direct Oblimin rotation was preferred due to the data being correlated. The PCA output for both physical fitness and CVH explained > 60% of the variance in each and was used in the multivariable regression models [50]. To control for collinearity the VIF and Durbin-Watson statistics were used. A VIF < 5 was used to indicate that no substantial collinearity was present and a Durbin-Watson statistic between 1.5 and 2.5 indicated no autocorrelation was present. Linear least absolute shrinkage and selection operator (LASSO) regression was also used to build a prediction model for each physical fitness and CVH parameter to reduce the number of predictors (*n* = 19). To ensure cross-validation of the model and evaluate the predictive ability of the model a five-fold cross-validation method was used. For reporting, the more parsimonious model within 1 standard error of the optimal model was preferred. Indicators (physical fitness and CVH) with non-zero coefficients were reported, only. For data that were not normally distributed, data were fractionally ranked, and then normalized using the inverse DF, IDF.NORMAL transformation [51]. A *p*-value of < 0.05 was used to indicate statistical significance.

## Results

In Table 1 we present data on all six occupational-specific tasks based on participant characteristics. Time to complete all occupational-specific tasks were significantly different between male and female firefighters (*p* < 0.001), with males performing better than females. Based on age-group, performance times of the individual occupational-specific tasks was significantly different between age categories (*p* < 0.001). Firefighters with good grip strength (*p* < 0.01), leg strength (*p* < 0.001), push-ups (*p* < 0.001) and sit-ups (*p* < 0.001) had significantly shorter completion times on all individual occupational-specific tasks. Aged firefighters had significantly longer completion times on all occupational-specific tasks (*p* < 0.01), except the forcible entry. Firefighters that were obese, had central obesity, and were physical inactive had significantly longer completion times for all the occupational-specific tasks (*p* < 0.01). Firefighters that reported UBMSIs had longer completion times on the step-up and ladder raise and extension tasks (*p* < 0.05). Firefighters that reported LoBMSIs had longer completion times on the step-up, charged hose drag and pull and the ladder raise and extension (*p* < 0.05), and firefighters with LoBMSD had longer completion times on the ladder raise and extension (*p* < 0.05).

**Table 1:** Completion time for individual occupational tasks according to sex, age-group, physical fitness, cardiovascular and musculoskeletal health

	Step-Up		Charged hose drag and pull		Forcible entry		Equipment carry		Ladder raise and extension		Rescue drag	
Variable	N	*X̃* (p25^th^—p75^th^)	N	*X̃* (p25^th^—p75^th^)	N	*X̃* (p25^th^—p75^th^)	N	*X̃* (p25^th^—p75^th^)	N	*X̃* (p25^th^—p75^th^)	N	*X̃* (p25^th^—p75^th^)
**Demographic characteristics**												
Total firefighters	279	65.0 (58.0, 75.0)	277	86.0 (66.0, 115.0)	273	32.0 (21.0, 48.5)	271	50.0 (40.0, 67.0)	268	72.5 (55.0, 96.8)	267	51.0 (38.0, 77.0)
Sex												
Male	248	65.0 (58.0, 74.0)	247	82.0 (65.0, 105.0)	244	29.6 (20.0, 46.0)	242	48.1 (38.0, 60.9)	239	70.0 (54.0, 89.0)	239	50.0 (37.0, 66.0)
Female	31	79 (72.0, 103.0)	30	172.5(120.3, 218.5)	29	54 (38.2, 100.0)	29	75 (57.5, 112.5)	29	75.0 (57.5, 112.5)	28	111.0 (78.8, 164.5)
*p*-value		< 0.001		< 0.001		< 0.001		< 0.001		< 0.001		< 0.001
**Age group**												
20–29 years	72	63.5 (56.0, 71.0)	72	80.4 (59.5, 99.8)	71	27.0 (20.0, 36.0)	71	46.0 (35.8, 55.0)	71	67.4 (55.0, 88.0)	71	48.0 (36.0, 62.0)
30–39 years	88	63 (57.0, 73.9)	88	79.0 (63.0, 106.8)	87	27.0 (19.0, 49.0)	87	47.0 (37.0, 58.0)	86	66.5 (54.0, 80.5)	87	48.0 (36.4, 69.3)
40–49 years	70	67.5 (61.0, 76.5)	70	107.0 (79.0, 148.5)	70	38.0 (26.0, 60.0)	68	57.5 (45.0, 73.8)	67	87.0 (59.0, 120.0)	66	56.5 (42.4, 89.3)
50 + years	48	73.9 (62.2, 84.8)	46	98.0 (79.5, 141.3)	44	37.5 (22.8, 53.8)	44	58.0 (44.0, 86.3)	43	82.0 (60.0, 102.0)	43	57.0 (46.9, 85.4)
*p*-value		< 0.001		< 0.001		0.004		< 0.001		0.001		0.002
**Physical fitness**												
Good relV̇O2max	144	65.0 (57.4, 72.9)	144	89.5 (68.0, 110.8)	142	31.5 (22.8, 48.0)	141	(50.0 (39.0, 66.5)	139	74.0 (55.0, 96.0)	138	51.0 (38.9, 78.0)
Low relV̇O2max	135	68.0 (60.0, 80.0)	133	82.6 (66.0, 121.4)	131	33.0 (19.0, 52.0)	130	51.9 (40.0, 71.0)	129	72 (54.6, 98.0)	129	50.0 (37.7, 77.0)
*p*-value		0.022		0.746		0.819		0.587		0.710		0.652
Good abV̇O2max	140	62.5 (56.0, 73.5)	139	70.0 (60.0, 91.2)	139	25.0 (18.0, 38.0)	139	44.0 (34.3, 54.0)	139	62.0 (51.2, 82.0)	139	44.0 (34.0, 58.0)
Low abV̇O2max	139	69.0 (62.0, 78.0)	138	104.0 (82.0, 141.3)	134	38.2 (27.0, 60.02)	132	58.5 (47.0, 82.8)	129	82.0 (64.0, 110.9)	128	61.0 (47.3, 89.8)
*p*-value		< 0.001		< 0.001		< 0.001		< 0.001		< 0.001		< 0.001
Good grip strength	121	62.7 (56.0, 73.9)	121	71.0 (59.0, 91.0)	121	25.6 (17.0, 39.5)	121	44.0 (33.0, 56.0)	121	60.0 (48.0, 83.5)	121	42.0 (33.4, 60.5)
Low leg strength	158	67.5 (61.0, 76.3)	156	100.0 (79.3, 139.0)	152	36.0 (25.0, 59.8)	150	56.0 (45.0, 75.2)	147	78.0 (64.0, 103.0)	146	55.3 (48.0, 85.5)
*p*-value		0.003		< 0.001		< 0.001		< 0.001		< 0.001		< 0.001
Good leg strength	107	61.0 (54.0, 72.0)	107	68.6 (58.0, 90.0)	107	25.0 (17.0, 35.0)	107	42.0 (33.0, 54.0)	107	59.0 (49.0, 79.0)	107	41.0 (32.0, 53.0)
Low leg strength	172	67.5 (61.0, 77.8)	170	99.0 (77.8, 141.3)	166	38.0 (25.0, 65.2)	164	56.1 (45.0, 78.5)	161	79.0 (61.5, 103.0)	160	57.5 (48.0, 87.8)
*p*-value		< 0.001		< 0.001		< 0.001		< 0.001		< 0.001		< 0.001
Good push-ups stamina	153	63.0 (56.0, 72.0)	153	77.0 (59.0, 100.0)	153	27.0 (20.0, 42.5)	151	45.0 (34.9, 56.0)	151	64.0 (51.0, 88.0)	150	43.7 (33.9, 58.3)
Low push-up stamina	126	70.0 (62.5, 80.0)	124	101.5 (80.2, 144.2)	120	38.0 (25.2, 60.0)	120	58.0 (47.4, 83.0)	117	80.0 (64.0, 103.0)	117	62.0 (50.0, 88.5)
*p*-value		< 0.001		< 0.001		< 0.001		< 0.001		< 0.001		< 0.001
Good sit-ups stamina	151	63.0 (56.0, 71.0)	153	80.9 (60.5, 101.0)	152	27.0 (19.0, 42.8)	151	46.0 (35.0, 59.0)	151	63.0 (49.8, 89.0)	151	48.0 (34.0, 64.0)
Low push-ups stamina	126	71.0 (61.8, 80.3)	124	100.0 (75.3, 141.3)	121	38.0 (25.0, 55.5)	120	38.0 (25.0, 55.5)	117	79.0 (67.2, 103.0)	116	57.0 (43.3, 85.8)
*p*-value		< 0.001		< 0.001		< 0.001		< 0.001		< 0.001		< 0.001
Good flexibility	148	65.0 (57.4, 75.0)	147	83.0 (64.0, 119.0)	147	29.1 (20.0, 48.0)	146	47.2 (37.0, 66.3)	146	70.0 (54.0, 96.0)	146	50.0 (37.0, 77.3)
Low flexibility	131	66.0 (60.0, 76.0)	130	88.9 (67.5, 111.3)	126	34.5 (21.8, 53.0)	125	53.2 (42.7, 72.0)	122	74.0 (57.8, 99.3)	121	53.0 (41.0, 77.5)
*p*-value		0.384		0.540		0.371		0.038		0.336		0.393
**Cardiovascular health**												
Aged	77	69.0 (62.9, 82.0)	75	102 (80.0, 135.0)	73	38.0 (25.5, 53.0)	72	58.0 (45.0, 82.9)	70	82 (60.0, 103.3)	70	56.5 (46.7, 82.1)
Young	202	64.0 (57.0, 74.0)	202	81.5 (62.9, 106.3)	200	30.0 (20.0, 48.0)	199	48.0 (37.0, 61.6)	198	70.0 (54.0, 92.0)	197	50.0 (35.6, 70.0)
*p*-value		< 0.001		< 0.001		0.062		< 0.001		0.004		0.003
Obesity	73	75 (63.0, 91.0)	71	102 (69.0, 157.7)	69	38.0 (25.0, 58.0)	69	58.0 (45.5, 86.6)	68	81.5 (60.5, 119.8)	67	57.0 (45.8, 90.2)
Normal	206	64.5 (57.0, 72.3)	206	83.5 (65.8, 107.3)	204	29.6 (20.0, 47.0)	202	48.1 (38.0, 62.3)	200	70.0 (53.2, 92.0)	200	50.0 (37.0, 69.8)
*p*-value		< 0.001		0.003		0.037		0.001		< 0.001		0.008
Central obesity	135	71 (63.0, 80.0)	133	93.0 (68.5.0, 141.5)	130	35.2 (22.5, 55.5)	130	55.0 (42.8, 79.2)	129	77.0 (59.0, 102.5)	128	55.2 (41.0, 85.8)
Normal	144	64.0 (57.0, 71.0)	144	82.0 (63.0, 103.4)	143	30.0 (20.0, 43.3	141	47.0 (37.0, 59.0)	139	68.0 (52.0, 90.0)	139	48.0 (37.0, 66.0)
*p*-value		< 0.001		0.002		0.112		< 0.001		0.003		0.006
Hypertension	128	66.5 (60.0, 77.5)	126	84.4 (66.0, 118.5)	124	31.2 (23.0, 47.0)	123	51.8 (40.0, 68.0)	122	71.5 (55.0, 00.3)	121	50.0 (27.5, 71.5)
Normal	151	65.0 (57.0, 75.)	151	88.6 (66.0, 115.0)	149	32.0 (20.0, 53.5)	148	50.0 (39.0, 67.0)	146	73.4 (55.0, 93.5)	146	51.0 (39.0, 78.5)
*p*-value		0.196		0.761		0.837		0.635		0.647		0.842
Dyslipidaemia	90	68.5 (60.0, 80.0)	89	93.0 (67.5, 134.5)	88	34.4 (22.7, 53.8)	87	55.0 (42.0, 84.0)	86	76.0 (55.8, 105.8)	86	53.5 (38.0, 86.3)
Normal	189	65.0 (58.0, 74.9)	188	83.5 (65.0, 107.8)	185	31.0 (20.0, 47.5)	184	49.7 (39.0, 61.9)	182	70.0 (54.8, 92.3)	184	50.6 (38.0, 70.0)
*p*-value		0.125		0.039		0.281		0.074		0.104		0.218
Diabetes	13	65.0 (60.0, 91.5)	12	108.9 (76.8, 138.3)	12	35.5 (26.8, 53.0)	12	56.5 (38.0, 69.0)	12	84.5 (63.5, 102.5)	12	56.0 (37.6, 92.8)
Normal	266	65.5 (58.0, 75.0)	265	86.0 (66.0, 114.5)	261	32.0 (21.0, 48.5)	259	50.0 (40.0, 67.0)	256	72.0 (55.0, 96.0)	255	50.6 (38.0, 77.0)
*p*-value		0.340		0.238		0.431		0.624		0.197		0.575
Physical inactivity	177	67.0 (60.0, 78.0)	176	95.0 (70.0, 133.5)	172	36.9 (24.0, 54.8)	170	55.0 (44.0, 74.0)	168	76.0 (59.0, 100.0)	167	55.0 (43.4, 83.0)
Active	102	63.0 (57.0, 73.0)	101	79.0 (61.0, 97.5)	101	26.0 (19.0, 36.5)	101	45.0 (34.4, 56.5)	100	63.0 (49.2, 84.8)	100	43.0 (33.9, 57.8)
*p*-value		0.018		< 0.001		< 0.001		< 0.001		< 0.001		< 0.001
Cigarette smoker	98	65.0 (60.0, 74.0)	98	85.4 (66.8, 110.0)	97	33.7 (22.3, 53.0)	97	51.8 (40.0, 67.0)	97	51.8 (40.0, 67.0)	97	54.0 (41.5, 74.0)
Non-Smoker	181	66.0 (58.0, 76.0)	179	86.0 (65.0, 118.0)	176	31.7 (20.3, 47.0)	174	50.0 (39.0, 67.3)	171	73.8 (53.0, 97.0)	170	50.0 (37.0, 77.3)
*p*-value		0.850		0.220		0.594		0.786		0.783		0.311
Poor diet	74	64.0 (57.2, 76.5)	73	82.0 (65.5, 112.5)	72	30.0 (21.0, 49.1)	71	50.0 (40.5, 58.0)	71	70.0 (57.8, 87.0)	71	50.0 (40.0, 65.1)
Normal	205	66.3 (59.5, 75.0)	204	87.0 (66.0, 115.8)	201	33.0 (21.3, 48.5)	200	50.0 (39.3, 73.0)	197	74.0 (54.6, 99.0)	196	51.0 (37.5, 78.0)
*p*-value		0.210		0.437		0.731		0.365		0.342		0.557
Good CVHI	85	71.0 (60.0, 80.0)	84	92.5 (69.1, 138.4)	83	36.0 (25.0, 55.0)	83	58.0 (44.0, 84.0)	83	78.0 (60.0, 102.0)	83	62.0 (42.0, 89.0)
Intermediate	160	64.3 (58.0, 74.9)	159	82.0 (63.0, 108.0)	156	28.5 (20.0, 45.5)	154	46.0 (36.8, 58.3)	151	69.0 (49.8, 91.0)	150	47.5 (34.0, 62.3)
Poor CVHI	32	66.0 (58.3, 72.8)	32	89.0 (79.5, 124.3)	32	34.5 (24.5, 68.0)	32	49.0 (45.3, 55.8)	32	78.0 (58.3, 102.0)	32	54.5 (44.0, 83.8)
*p*-value		0.105		0.014		0.028		< 0.001		0.003		< 0.001
**Musculoskeletal health**												
MSI	116	69.0 (60.3, 78.0)	114	85.0 (65.0, 124.5)	111	35.0 (21.0, 55.0)	110	53.0 (38.0, 71.0)	110	74.5 (55.0, 100.0)	110	55.0 (37.8, 83.0)
No MSI	162	64.8 (57.2, 74.0)	162	86.5 (66.0, 110.3)	161	31.0 (21.0, 46.0)	160	50.0 (42.0, 66.0)	157	71.0 (55.0, 94.0)	156	50.0 (38.1, 69.8)
*p*-value		0.018		0.371		0.362		0.711		0.394		0.143
UBMSI	58	70.0 (60.8, 79.3)	56	94.0 (75.3, 136.2)	54	76.5 (59.0, 102.3)	54	56.1 (42.5, 79.7)	55	38.0 (24.0, 60.0)	54	57.5 (38.6, 86.8)
No UBMSI	221	65.0 (58.0, 74.9)	221	86.0 (65.5, 111.5)	214	71.0 (55.0, 95.0)	217	49.0 (40.0, 66.0)	218	30.7 (20.0, 47.0)	213	50.0 (38.0, 70.0)
*p*-value		0.043		0.105		0.216		0.103		0.035		0.085
LBMSI	65	69.0 (61.0, 78.5)	65	90.0 (63.5, 125.5)	63	76.0 (52.0, 100.0)	63	49.0 (37.0, 68.0)	63	31.3 (20.0, 47.0)	63	54.0 (35.0, 78.0)
No LBMSI	214	65.0 (58.0, 75.0)	212	85.5 (66.0, 110.8)	205	51.0 (55.0, 95.5)	208	51.0 (41.1, 67.0)	210	32.0 (21.5, 52.3)	204	50.6 (39.0, 76.5)
*p*-value		0.074		0.482		0.597		0.380		0.742		0.943
LoBMSI	22	73.3 (64.5, 87.5)	21	108.0 (80.5, 144.5)	20	91.5 (59.3, 118.5)	20	60.3 (36.3, 87.8)	21	46.0 (27.0, 60.0)	20	63.5 (39.0, 95.0)
No LoBMSI	257	65.0 (58.0, 75.0)	256	84.4 (66.0, 112.0)	248	71.0 (55.0, 95.0)	251	50.0 (40.0, 66.0)	252	31.2 (20.3, 47.0)	247	50.6 (38.0, 74.3)
*p*-value		0.010		0.013		0.083		0.279		0.027		0.112
MSD	116	66.0 (58.1, 75.8)	115	86.0 (65.0, 118.0)	113	34.0 (21.8, 53.0)	111	51.7 (39.0, 68.0)	111	70.6 (55.0, 100.0)	110	53.0 (37.8, 81.0)
No MSD	163	65.0 (58.0, 75.0)	162	86.0 (66.9, 115.0)	160	30.2 (20.3, 48.0)	160	50.0 (40.0, 67.0)	157	74.0 (55.0, 94.0)	157	50.0 (38.2, 71.5)
*p*-value		0.884		0.916		0.333		0.969		0.956		0.458
UBMSD	99	65.0 (57.3, 78.0)	98	85.0 (65.0, 116.3)	97	34.4 (21.3, 54.5)	96	52.5 (39.3, 70.3)	96	70.0 (53.3, 99.5)	95	55.0 (38.0, 81.0)
No UBMSD	180	65.5 (59.0, 75.0)	179	86.0 (66.9, 115.0)	176	30.7 (21.0, 46.0)	175	50.0 (40.0, 67.0)	172	74.0 (57.0, 95.8)	172	50.0 (38.1, 73.9)
*p*-value		0.933		0.891		0.287		0.287		0.640		0.260
LBMSD	67	65.0 (57.0, 75.0)	67	82.0 (62.4, 118.0)	65	32.0 (21.3, 43.0)	64	48.0 (37.5, 62.3)	64	65.0 (50.3, 96.5)	64	49.8 (34.3, 81.0)
No LBMSD	212	66.0 (58.6, 76.0)	210	87.9 (67.8, 115.0)	208	32.0 (21.0, 51.8)	207	51.8 (40.0, 68.0)	204	74.5 (57.1, 96.8)	203	51.0 (39.0, 74.3)
*p*-value		0.476		0.337		0.773		0.382		0.123		0.560
LoBMSD	59	68.0 (58.4, 80.0)	59	90.0 (65.0, 136.6)	57	75.0 (54.0, 108.4)	57	53.2 (40.0, 79.3)	58	38.0 (23.8, 63.5)	56	57.5 (37.5, 95.8)
No LoBMSD	220	65.0 (58.0, 75.0)	218	86.0 (66.0, 112.5)	211	72.0 (55.0, 96.0)	214	50.0 (39.8, 66.0)	215	31.0 (20.0, 46.0)	211	50.0 (38.0, 70.0)
*p*-value		0.302		0.362		0.699		0.287		0.046		0.093

*MSI* Musculoskeletal injury, *UBMSI* Upper body musculoskeletal injury, *LBMSI* Lower body musculoskeletal injury, *LoBMSI* Lower back musculoskeletal injury, *MSD* Musculoskeletal discomfort, *UBMSD* Upper body musculoskeletal discomfort, *LBMSD* Lower body musculoskeletal discomfort, *LoBMSD* Lower back musculoskeletal injury

In Table 2 we indicate the association between demographic characteristics, physical fitness, cardiovascular health and occupational-specific task performance. Multivariable analyses indicated that an increase in abV̇O2max was associated with a shorter completion time for the step-up, charged hose drag and pull, forcible entry, equipment carry, ladder raise and extension and the rescue drag completion times. An increase in grip and leg strength was associated with a shorter completion time for the charged hose drag and pull, forcible entry, and equipment carry. In addition, grip strength was associated with shorter ladder raise and extension and rescue drag completion times. An increase in push-ups and sit-ups capacity was associated with a shorter completion time for the step-up, charged hose drag and pull, forcible entry, equipment carry, forcible entry and rescue drag. An increase in LBM was associated with a shorter completion time in the charged hose drag and pull, forcible entry, equipment carry and rescue drag tasks.

**Table 2:** Linear associations between physical fitness, cardiovascular and musculoskeletal health and occupational-specific task performance in firefighters

	Step-up		Charged hose drag and pull		Forcible entry		Equipment carry		Ladder raise and extension		Rescue drag	
	Model 1 ^a^	Model 2 ^b^	Model 1 ^a^	Model 2 ^b^	Model 1 ^a^	Model 2 ^b^	Model 1^a^	Model 2 ^b^	Model 1 ^a^	Model 2 ^b^	Model 1 ^a^	Model 2^b^
	β (R^2^)	β (R^2^)	β (R^2^)	β (R^2^)	β (R^2^)	β (R^2^)	β (R^2^)	β (R^2^)	β (R^2^)	β (R^2^)	β (R^2^)	β (R^2^)
Model: Demographics												
Age (years)	0.25 (0.06)^e^	0.25 (0.21)^e^	0.32 (0.09)^e^	0.32 (0.45)^e^	0.17 (0.03)^d^	0.16 (0.22)^d^	0.30 (0.09)^e^	0.28 (0.37)^e^	0.17 (0.03)^d^	0.16 (0.22)^d^	0.27 (0.07)^e^	0.26 (0.36)^e^
YoE (years)	0.27 (0.07)^e^	0.44 (0.25)^e^	0.29 (0.08)^e^	0.29 (0.47)^d^	0.15 (0.02)^d^	0.14 (0.23)	0.28 ^c^0.08)	0.29 (0.39)^c^	0.15 (0.02)^d^	0.14 (0.23)	0.22 (0.05)^e^	0.14 (0.36)
Weight (kg)	0.04 (0.00)	0.15 (0.23)^c^	-0.17 (0.03)^e^	-0.07 (0.45)	-0.21 (0.04)^e^	-0.13 (0.24)^c^	-0.12 (0.01)	-0.03 (0.37)	-0.21 (0.04)^e^	-0.13 (0.24)^c^	-0.19 (0.04)^d^	-0.12 (0.37)
Height (cm)	-0.34 (0.12)^e^	-0.23 (0.21)^e^	-0.54 (0.29)^d^	-0.43 (0.45)^e^	-0.38 (0.14)^e^	-0.30 (0.22)^e^	-0.45 (0.19)^e^	-0.35 (0.37)	-0.38 (0.14)^e^	-0.30 (0.22)^e^	-0.46 (0.21)^e^	-0.35 (0.36)^e^
Model: Physical fitness												
V̇O2max (L•min)	-0.22 (0.05)^e^	-0.22 (21)^d^	-0.44 (0.19)^e^	-0.17 (0.47)^e^	-0.38 (0.14)^e^	-0.22 (0.27)^e^	-0.43 (0.19)^e^	-0.18 (0.39)^d^	-0.38 (0.14)^e^	-0.22 (0.27)^e^	-0.43 (0.18)^e^	-0.19 (0.38)^e^
V̇O2max (mL•kg•min)	-0.14 (0.02)^c^	-0.19 (0.24)^d^	0.01 (0.00)	-	0.07 (0.01)	-	-0.05 (0.00)	-	0.07 (0.01)	-	0.02 (0.00)	-
Grip strength (kg)	-0.31 (0.09)^e^	-0.10 (0.22)	-0.53 (0.28)^e^	-0.27 (0.49)^e^	-0.37 (0.14)^e^	-0.20 (0.25)^d^	-0.43 (0.19)^e^	-0.22 (0.40)^e^	-0.37 (0.14)^e^	-0.20 (0.25)^d^	-0.50 (0.25)^e^	-0.31 (0.42)^e^
Leg strength (kg)	-0.31 (0.09)^e^	-0.11 (0.22)	-0.52 (0.27)^e^	-0.26 (0.50)^e^	-0.39 (0.16)^e^	-0.23 (0.26)^e^	-0.43 (0.19)^e^	-0.18 (0.39)^d^	-0.39 (0.16)^e^	-0.23 (0.26)^e^	-0.49 (0.24)^e^	0.26 (0.41)
Push-ups (rpm)	-0.33 (0.11)^e^	0.25 (0.26)^e^	-0.44 (0.19)^e^	-0.35 (0.54)^e^	-0.29 (0.09)^e^	0.24 (0.27)^e^	-0.42 (0.17)^e^	-0.32 (0.45)^e^	-0.29 (0.09)^e^	0.24 (0.27)^e^	-0.41 (0.17)^e^	-0.32 (0.44)^e^
Sit-ups (rpm)	-0.41 (0.17)^e^	0.09 (0.31)^e^	-0.39 (0.16)^e^	-0.28 (0.51)^e^	-0.26 (0.07)^e^	-0.21 (0.26)^e^	-0.34 (0.12)^e^	-0.24 (0.42)^e^	-0.26 (0.07)^e^	-0.21 (0.26)^e^	-0.35 (0.12)^e^	-0.25 (0.41)^e^
Sit-and-reach (cm)	-0.09 (0.01)	-0.12 (0.23)^c^	-0.04 (0.00)	-0.05 (0.45)	-0.04 (0.00)^e^	-0.05 (0.22)	-0.13 (0.02)		-0.04 (0.00)^e^	-0.05 (0.22)	-0.07 (0.01)	
Lean body Mass (kg)	-0.27 (0.07)^e^	-0.08 (0.22)	-0.44 (0.19)^e^	-0.18 (0.46)^d^	-0.38 (0.14)^e^	-0.27 (0.26)^e^	-0.37 (0.14)^e^	-0.18 (0.39)^c^	-0.38 (0.14)^e^	-0.27 (0.26)^e^	-0.43 (0.18)^e^	-0.27 (0.39)^e^
Model: Cardiovascular health												
Body mass index (kg•m^−2^)	0.23 (0.06)^e^	0.13 (0.22)^c^	0.11 (0.01)		-0.01 (0.00)		0.12 (0.01)		-0.01 (0.00)		0.06 (0.00)	
Bodyfat percentage (%)	0.35 (0.12)^e^	0.16 (0.23)^d^	0.29 (0.08)^e^	-0.03 (0.45)	0.14 (0.02)^c^	-0.04 (0.22)	0.28 (0.08)^e^	0.05 (0.37)	0.14 (0.02)^c^	-0.04 (0.22)	0.26 (0.07)^e^	-0.01 (0.36)
Waist circumference (cm)	0.16 (0.03)^d^	0.26 (0.23)^c^	0.05 (0.00)	-	-0.02 (0.00)	-	0.12 (0.02)	-	-0.02 (0.00)	-	0.03 (0.00)	-
SBP (mmHg)	-0.01 (0.00)	-	-0.14 (0.02)^c^	-0.09 (0.45)^c^	-0.09 (0.01)	-	-0.03 (0.00)	-	-0.09 (0.01)	-	-0.07 (0.01)	-
DBP (mmHg)	0.21 (0.04)^e^	0.13 (23)^c^	0.11 (0.01)	-	0.06 (0.00)	-	0.18 (0.03)	-	0.06 (0.00)	-	0.08 (0.01)	-
TC (mmol•L^−1^)	0.07 (0.00)	-	0.09 (0.01)	-	0.02 (0.00)	-	0.07 (0.01)	-	0.02 (0.00)	-	0.03 (0.00)	-
LDL-C (mmol•L^−1^)	0.05 (0.00)	-	0.08 (0.01)	-	0.05 (0.00)	-	0.06 (0.00)	-	0.05 (0.00)	-	0.02 (0.00)	-
HDL-C (mmol•L^−1^)	-0.06 (0.00)	-	0.04 (0.00)	-	-0.01 (0.00)	-	-0.01 (0.00)	-	-0.01 (0.00)	-	0.02 (0.00)	-
Triglycerides (mmol•L^−1^)	0.13 (0.02)^c^	0.11 (0.23)	0.07 (0.00)	-	0.02 (0.00)	-	0.09 (0.01)	-	0.02 (0.00)	-	0.07 (0.01)	-
NFBG (mmol•L^−1^)	0.03 (0.00)	-	-0.00 (0.00)	-	-0.05 (0.00)	-	0.02 (0.00)	-	-0.05 (0.00)	-	0.04 (0.00)	-
MET minutes (min)	-0.07 (0.01)	-	-0.18 (0.03)^d^	-0.11 (0.45)^c^	-0.23 (0.05)^e^	-0.19 (0.22)^e^	-0.29(0.09)^e^	-0.23 (0.37)^e^	-0.23 (0.05)^e^	-0.19 (0.22)^e^	-0.24 (0.06)^e^	-0.18 (0.36)^e^
Framingham risk score	0.14 (0.02)^c^	0.09 (0.22)	0.19 (0.04)^d^	0.12 (0.45)	0.09 (0.01)	-	0.19 (0.04)^e^	0.07 (0.37)	0.09 (0.01)	-	0.15 (0.02)^c^	0.07 (0.36)
Model: Heart rate variability												
Heart rate variability (ms)	-0.17 (0.03)^d^	-0.13 (0.23)^c^	-0.17 (0.03)^d^	-0.09 (0.46)	-0.16 (0.02)^c^	-0.08 (0.22)	-0.24 (0.06)^e^	0.14 (0.39)^d^	-0.16 (0.02)^c^	-0.08 (0.22)	-0.19 (0.03)^d^	-0.09 (0.36)
SDNN (ms)	-0.27 (0.07)^e^	-0.18 (25)^d^	-0.28 (0.08)^e^	-0.14 (0.46)^d^	-0.15 (0.02)^c^	-0.04 (0.22)	-0.33 (0.11)^e^	-0.17 (0.39)^d^	-0.15 (0.02)^c^	-0.04 (0.22)	-0.22 (0.05)^e^	-0.07 (0.35)
RMSSD (ms)	-0.21 (0.05)^e^	-0.15 (0.24)^d^	-0.21 (0.05)^e^	-0.10 (0.45)^c^	-0.09 (0.01)	-	-0.29 (0.08)^e^	-0.16 (0.29)	-0.09 (0.01)	-	-0.17 (0.03)^d^	0.05 (0.35)
LF (Hz)	-0.122 (0.02)^e^	-0.02 (0.22)	-0.19 (0.04)^d^	-0.05 (0.46)	-0.09 (0.01)	-	-0.16 (0.02)	-	-0.09 (0.01)	-	-0.15 (0.02)^c^	-0.02 (0.36)
HF (Hz)	-0.01 (0.00)	-	0.11 (0.01)	-	0.07 (0.00)	-	0.02 (0.00)	-	0.07 (0.00)	-	0.07 (0.01)	-
LF/HF (Hz)	-0.01 (0.00)	-	-0.01 (0.00)	-	-0.06 (0.00)	-	0.07 (0.01)	-	-0.06 (0.00)	-	-0.00 (0.00)	-

*YoE* Years of experience, *kg•m*^*−2*^ Kilogram per meter squared, *cm* Centimetre, *%* Percentage, *mm Hg* Millimetres of mercury, *mmol•L*^*−1*^ Millimole per litre, *MET* Metabolic equivalents, *ms* Millisecond, *Hz* Hertz, *BMI* Body mass index, *WC* Waist circumference, *SBP* Systolic blood pressure, *DBP* Diastolic blood pressure, *NFBG* Non-fasting blood glucose, *TC* Total cholesterol, *LDL-C* Low-density lipoprotein, *HDL-C* High-density lipoprotein, *SDNN* Standard deviation of all normal-to-normal, *RMSSD* Root-mean-square of successive differences, *LF* Low-frequency, *HF* High frequency, *LF/HF* Low and high frequency ratio, *rpm* Repetitions per minute

^a^Univariable models using linear regression

^b^Multivariable linear regression adjusted for covariates: age, sex, height and weekly metabolic equivalent minutes

^c^Indicates statistical significance < 0.05

^d^Indicates statistical significance < 0.01

^e^Indicates statistical significance < 0.001

For CVH, in the multivariable analyses, an increase in age was associated with an increase in the completion times of the step-up, charged hose drag and pull, ladder raise and extension, equipment carry, forcible entry and the rescue drag. An increase in height was associated with a decrease in completion times for the step-up, charged hose drag and pull, ladder raise and extension, equipment carry, forcible entry and the rescue drag. An increase in BMI and BF% was associated with an increase in the step-up completion time, only. An increase in SBP was associated with a shorter completion time in the charged hose drag and pull, only. An increase in weekly MET minutes was associated with a shorter completion time in the charged hose drag and pull, forcible entry, equipment carry and rescue drag, respectively. An increase in HRV, SDNN and RMSSD was associated with shorter completion times for all occupational-specific tasks (all *p* < 0.01). After adjustment for age, sex, height and weekly MET minutes, HRV and SDNN remained significantly associated with shorter completion times for all occupational-specific tasks.

In Table 3 we further delineate the interrelationships between physical fitness, cardiovascular health and occupational-specific task performance. For physical fitness, after adjustment for CVH and MSH, abV̇O_2max,_ abV̇O_2max,_ grip strength, leg strength, sit-ups and LBM remained significantly associated with all tasks (all *p* < 0.01). Push-ups capacity remained significantly associated with all tasks, except the step-up (all *p* < 0.001). Based on CVH, after adjustment for physical fitness and MSH, an increase in age was associated with slower completion times in the charged hose drag and pull, equipment carry and the rescue drag tasks. An increase in BMI was associated with slower completion times in the charged hose and pull (*p* < 0.01) and the ladder raise and extension (*p* < 0.01). An increase in DBP was associated with slower completion times in the step-up (*p* < 0.05) and equipment carry (*p* < 0.05). Framingham risk score was associated with slower completion times in the charged hose drag and pull (*p* < 0.001), equipment carry (*p* < 0.01) and rescue drag task (*p* < 0.01). In Model 3, an increase in SDNN and RMSSD was associated with faster completion times in the step-up (*p* < 0.05) and for the equipment carry and increase HRV, SDNN and RMSSD were associated with faster completion times (all *p* < 0.05).

**Table 3:** Multivariable linear associations between physical fitness, cardiovascular and musculoskeletal health and occupational-specific task performance in firefighters

	Step-up		Charged hose drag and pull		Forcible entry		Equipment carry		Ladder raise and extension		Rescue drag	
	Model 1^a^	Model 2^b^	Model 1^a^	Model 2^b^	Model 1^a^	Model 2^b^	Model 1^a^	Model 2^b^	Model 1^a^	Model 2^b^	Model 1^a^	Model 2^b^
	β (R^2^)	β (R^2^)	β (R^2^)	β (R^2^)	β (R^2^)	β (R^2^)	β (R^2^)	β (R^2^)	β (R^2^)	β (R^2^)	β (R^2^)	β (R^2^)
Model: Physical fitness												
V̇O2max (L•min)	-0.23 (0.16)^f^	-	-0.45 (0.28)^f^	-	-0.39 (0.17)^f^	-	-0.45 (0.28)^f^	-	0.38 (0.20)^f^	-	-0.45 (0.27)^f^	-
V̇O2max (mL•kg•min)	0.23 (0.13)^d^	-	0.82 (0.18)^f^	-	0.43 (0.10)^f^	-	0.39 (0.14)^f^	-	0.36 (0.12)^f^	-	0.48 (0.17)^f^	-
Grip strength (kg)	-0.29 (0.19)^f^	-	-0.49 (0.33)^f^	-	-0.35 (0.15)^f^	-	-0.42 (0.25)^f^	-	-0.45 (0.27)^f^	-	-0.48 (0.30)^f^	-
Leg strength (kg)	-0.27 (0.17)^f^	-	-0.50 (0.31)^f^	-	-0.39 (0.17)^f^	-	-0.42 (0.24)^f^	-	-0.24 (0.23)^f^	-	-0.46 (0.27)^f^	-
Push-ups (rpm)	-0.21 (0.12)	-	-0.58 (0.19)^f^	-	-0.45 (0.09)^f^	-	-0.49 (0.17)^f^	-	-0.35 (0.11)^f^	-	-0.57 (0.19)^f^	-
Sit-ups (rpm)	-0.46 (0.18)^f^	-	-0.49 (0.16)^f^	-	-0.37 (0.07)^f^	-	-0.30 (0.11)^e^	-	-044 (0.13)^f^	-	-0.40 (0.13)^f^	-
Sit-and-reach (cm)	0.09 (0.11)	-	-0.15 (0.09)^d^	-	0.06 (0.03)	-	0.03 (0.08)	-	0.06 (0.07)	-	0.09 (0.08)	-
Lean body Mass (kg)	-0.49 (0.29)^f^	-	-0.67 (0.43)^f^	-	-0.55 (0.26)^f^	-	-0.62 (0.38)^f^	-	-0.62 (0.36)^f^	-	-066 (0.41)^f^	-
Model: Cardiovascular health												
Age	-	0.06 (0.29)	-	0.14 (0.54)^e^	-	0.06 (0.28)	-	0.15 (0.42)^e^	-	0.03 90.41)	-	0.12 (0.47)^d^
Body mass index (kg•m^−2^)	-	0.32 (0.29)	-	0.41 (0.54)^e^	-	0.18 (0.28)	-	0.11 (0.40)	-	0.41 (0.43)^e^	-	0.09 (0.46)
Bodyfat percentage (%)	-	-0.12 (0.29)	-	-0.27 (0.54)^e^	-	-0.23 (0.29)	-	-0.28 (0.42)^d^	-	-.012 (0.42)	-	-0.27 (0.48)^d^
Waist circumference (cm)	-	0.06 (0.29)	-	0.17 (0.53)^d^	-	0.19 (0.29)	-	0.24 (0.43)^f^	-	0.11 (0.42)	-	0.24 (0.48)^e^
SBP (mmHg)	-	0.03 (0.29)	-	-0.02 (0.52)	-	0.02 (0.28)	-	0.08 (0.41)	-	0.02 (0.41)	-	0.07 (0.47)
DBP (mmHg)	-	0.12 (0.29)^d^	-	0.06 (0.52)	-	0.05 (0.28)	-	0.12 (0.42)^d^	-	0.06 (0.42)	-	0.04 (0.46)
TC (mmol•L^−1^)	-	0.05 (0.29)	-	0.09 (0.53)^d^	-	0.03 (0.28)	-	0.08 (0.41)	-	0.09 (0.42)	-	0.03 (0.46)
LDL-C (mmol•L^−1^)	-	0.05 (0.29)	-	0.09 (0.53)^d^	-	0.06 (0.28)	-	0.07 (0.41)	-	0.08 (0.42)	-	0.03 (0.46)
HDL-C (mmol•L^−1^)	-	-0.10 (0.29)	-	-0.06 (0.53)	-	-0.11 (0.29)^d^	-	-0.09 (0.41)	-	-0.06 (0.42)	-	-0.09 (0.47)^d^
Triglycerides (mmol•L^−1^)	-	0.05 (0.29)	-	0.04 (0.52)	-	0.03 (0.28)	-	0.07 (0.41)	-	0.04 (0.42)	-	0.05 (0.47)
NFBG (mmol•L^−1^)	-	-0.08 (0.29)	-	-0.07 (0.53)	-	-0.08 (0.29)	-	-0.05 (0.40)	-	-0.05 (0.42)	-	-0.00 (0.46)
MET minutes (min)	-	0.00 (0.29)	-	-0.08 (0.53)	-	-0.18 (0.31)^f^	-	-0.21 (0.45)^f^	-	-0.11 (0.43)^d^	-	-0.13 (-0.48)^e^
Framingham risk score	-	0.07 (0.29)	-	0.15 (0.54)^f^	-	0.09 (0.29)	-	0.16 (0.42)^e^	-	0.08 (0.42)	-	0.11 (0.47)^d^
	Model 3^c^		Model 3^c^		Model 3^c^		Model 3^c^		Model 3^c^		Model 3^c^	
	OR (95% CI)		OR (95% CI)		OR (95% CI)		OR (95% CI)		OR (95% CI)		OR (95% CI)	
Model: Heart rate variability												
Heart rate variability (ms)	-0.06 (0.29)		-0.02 (0.53)		-0.06 (0.29)		-0.10 (0.43)^d^		-0.09 (0.43)		-0.07 (0.48)	
SDNN (ms)	-0.12(0.30)^d^		-0.04 (0.53)		0.02 (0.29)		-0.12 (0.43)^d^		-0.06 (0.41)		-0.01 (0,47)	
RMSSD (ms)	-013. (0.30)^d^		0.04 (0.53)		-0.03 (0.29)		0.13 (0.44)^d^		-0.05 (0.41)		-0.02 (0.47)	
LF (Hz)	0.03 (0.30)		-0.03 (0.53)		-0.03 (0.29)		-0.01 (0.43)		-0.03 (0.43)		0.01 (0.48)	
HF (Hz)	-0.06 (0.31)		-0.00 (0.55)		-0.02 (0.29)		-0.08 (0.44)		-0.02 (0.43)		-0.03 (0.48)	
LF/HF (Hz)	-0.22 (0.31)		-0.02 (0.55)		-0.04 (0.29)		-0.08 (0.44)		-0.02 (0.43)		-0.01 (0.49)	

*kg•m*^*−2*^ Kilogram per meter squared, *cm* Centimetre, *%* Percentage, *mm Hg* Millimetres of mercury, *mmol•L*^*−1*^ Millimole per litre, *MET* Metabolic equivalents, *ms* Millisecond, *Hz* Hertz, *BMI* Body mass index, *WC* Waist circumference, *SBP* Systolic blood pressure, *DBP* Diastolic blood pressure, *NFBG* Non-fasting blood glucose, *TC* Total cholesterol, *LDL-C* Low-density lipoprotein, *HDL-C* High-density lipoprotein, *SDNN* Standard deviation of all normal-to-normal, *RMSSD* Root-mean-square of successive differences, *LF* Low-frequency, *HF* High frequency, *LF/HF* Low and high frequency ratio, *rpm* Repetitions per minute

^a^Multivariable linear regression adjusted for covariates: cardiovascular health and musculoskeletal health

^b^Multivariable linear regression adjusted for covariates: physical fitness and musculoskeletal health

^c^Multivariable linear regression adjusted for covariates: physical fitness and cardiovascular health

^d^Indicates statistical significance < 0.05

^e^Indicates statistical significance < 0.01

^f^Indicates statistical significance < 0.001

In Table 4, multivariable analysis is conducted to determine the association between we between physical fitness, cardiovascular health, musculoskeletal health and occupational-specific task performance, controlling for all covariates. Based on physical fitness, multivariable analysis in Model 1 showed that an increase in abV̇O2max remained significantly associated with faster completion times in the step-up, charged hose drag and pull, forcible entry, equipment carry, ladder raise and extension and rescue drag, and relV̇O2max remained significantly associated with the step-up task. An increase in grip strength was associated with faster completion times of the charged hose drag and pull, forcible entry, equipment carry, ladder raise and extension and the rescue drag task. Leg strength was associated with faster completion times in all tasks. Increased push-ups capacity was associated with faster completion times for all tasks (all *p* < 0.01), except the step-up. An increase in sit-ups capacity was associated with a decrease in completion times in the step-up, charged hose drag and pull, forcible entry and rescue drag tasks. Lean body mass was associated with a decrease in the completion in all tasks, except the step-up task. Based on CVH, in Model 2, an increase in BMI was associated with a decrease in completion times of the step-up and charged hose drag and pull. An increase in BF% was associated with faster completion times for the forcible entry, equipment carry and rescue drag tasks. In Model 3, an increase in SDNN and RMSSD was associated with a decrease in completion time of the step-up and an increase in HRV and RMSSD remained associated with faster completion times in the equipment carry task.

**Table 4:** Linear associations between physical fitness, cardiovascular and musculoskeletal health and occupational-specific task performance in firefighters

	Step-up		Charged hose drag and pull		Forcible entry		Equipment carry		Ladder raise and extension		Rescue drag	
	Model 1^a^	Model 2^b^	Model 1^a^	Model 2^b^	Model 1^a^	Model 2^b^	Model 1^a^	Model 2^b^	Model 1^a^	Model 2^b^	Model 1^a^	Model 2^b^
	β (R^2^)	β (R^2^)	β (R^2^)	β (R^2^)	β (R^2^)	β (R^2^)	β (R^2^)	β (R^2^)	β (R^2^)	β (R^2^)	β (R^2^)	β (R^2^)
Model: Physical fitness												
V̇O2max (L•min)	-0.26 (0.24)^f^	-	-0.22 (0.50)^f^	-	-0.27 (0.28)^f^	-	-0.27 (0.45)^f^	-	-0.19 (0.40)^e^	-	-0.28 (0.42)^f^	-
V̇O2max (mL•kg•min)	-0.27 (0.26)^f^	-	0.33 (0.51)^f^	-	0.30 (0.27)^f^	-	0.29 (0.42)^e^	-	0.17 (0.39)^d^	-	0.35 (0.42)^f^	-
Grip strength (kg)	0.12 (0.29)	-	-0.25 (0.52)^f^	-	-0.19 (0.26)^e^	-	-0.21 (0.43)^f^	-	-0.23 (0.42)^f^	-	-0.31 (0.44)^f^	-
Leg strength (kg)	-0.13 (0.28)^d^	-	-0.28 (0.53)^f^	-	-0.23 (0.27)^f^	-	-0.19 (0.43)^f^	-	-0.22 (0.41)^f^	-	-0.28 (0.43)^f^	-
Push-ups (rpm)	0.03 (0.28)	-	-0.37 (0.52)^f^	-	-0.29 (0.26)^e^	-	-0.29 (0.43)^f^	-	-0.29 (0.43)^f^	-	-0.38 (0.42)^f^	-
Sit-ups (rpm)	-0.27 (0.29)^e^	-	0.22 (0.49)^e^	-	-0.22 (0.25)^d^	-	0.09 (0.41)	-	0.10 (0.38)	-	-0.21 (0.38)^d^	-
Sit-and-reach (cm)	-0.05 (0.28)	-	0.13 (0.49)^d^	-	0.06 (0.24)	-	-0.02 (0.41)	-	-0.04 (0.38)	-	-0.07 (0.37)	-
Lean body Mass (kg)	-0.01 (0.28)	-	-0.37 (0.53)^f^	-	-0.42 (0.30)^f^	-	-0.37 (0.47)^f^	-	-0.37 (0.47)^f^	-	-0.49 (0.47)^f^	-
Model: Cardiovascular health												
Body mass index (kg•m^−2^)	-	-0.18 (0.29)^d^	-	-0.17 (0.49)^e^	-		-	-	-	-	-	-
Bodyfat percentage (%)	-	-0.18 (0.29)	-	-	-	-0.29 (0.25)^e^	-	-0.21 (0.40)^e^	-	-	-	-0.26 (0.37)^e^
Waist circumference (cm)	-	-0.13 (0.29)	-	-	-	-	-	-	-	-	-	-
SBP (mmHg)	-	-	-	-	-	-	-	-	-	-	-	-
DBP (mmHg)	-	0.04 (0.29)	-	-	-	-	-	-	-	-	-	-
TC (mmol•L^−1^)	-	-	-	-	-	-	-	-	-	-	-	-
LDL-C (mmol•L^−1^)	-	-	-	-	-	-	-	-	-	-	-	-
HDL-C (mmol•L^−1^)	-	-	-	-	-	-	-	-	-	-	-	-
Triglycerides (mmol•L^−1^)	-	-	-	-	-	-	-	-	-	-	-	-
NFBG (mmol•L^−1^)	-	-	-	-	-	-	-	-	-	-	-	-
MET minutes (min)	-	-	-	-0.10 (0.47)^d^	-	-0.20 (0.23)^e^	-	-0.24 (0.39)^f^	-	-	-	0.19 (0.35)^f^
Framingham risk score	-	0.01 (0.29)	-	0.11 (0.47)	-	-	-	0.01 (0.39)	-	-	-	0.02 (0.35)
	Model 3^c^		Model 3^c^		Model 3^c^		Model 3^c^		Model 3^c^		Model 3^c^	
	OR (95% CI)		OR (95% CI)		OR (95% CI)		OR (95% CI)		OR (95% CI)		OR (95% CI)	
Model: Heart rate variability												
Heart rate variability (ms)	-0.10 (0.33)	-	-0.04 (0.58)	-	-0.06 (0.31)	-	-0.11 (0.47)^d^	-	-0.09 (0.45)	-	-0.06 (0.48)	-
SDNN (ms)	-0.12 (0.33)^d^	-	-0.04 (0.58)	-	0.05 (0.31)	-	-0.09 (0.47)	-	-0.05 (0.44)	-	-0.01 (0,46)	-
RMSSD (ms)	-0.11 (0.33)^d^	-	-0.04 (0.58)	-	0.06 (0.31)	-	-0.12 (0.47)^d^	-	-0.05 (0.44)	-	-0.02 (0.47)	-
LF (Hz)	-	-	-0.02 (0.31)	-	-	-	-	-	-	-	0.01 (0.47)	-
HF (Hz)	-	-	-	-	-	-	-	-	-	-	-	-
LF/HF (Hz)	-	-	-	-	-	-	-	-	-	-	-	-

*kg•m*^*−2*^ Kilogram per meter squared, *cm* Centimetre, *%* Percentage, *mm Hg* Millimetres of mercury, *mmol•L*^*−1*^ Millimole per litre, *MET* Metabolic equivalents, *ms* Millisecond, *Hz* Hertz, *BMI* Body mass index, *WC* Waist circumference, *SBP* Systolic blood pressure, *DBP* Diastolic blood pressure, *NFBG* Non-fasting blood glucose, *TC* Total cholesterol, *LDL-C* Low-density lipoprotein, *HDL-C* High-density lipoprotein, *SDNN* Standard deviation of all normal-to-normal, *RMSSD* Root-mean-square of successive differences, *LF* Low-frequency, *HF* High frequency, *LF/HF* Low and high frequency ratio, *rpm* Repetitions per minute

^a^Multivariable linear regression adjusted for covariates: age, sex, height, weekly metabolic equivalents, cardiovascular health and musculoskeletal health

^b^Multivariable linear regression adjusted for covariates: age, sex, height, weekly metabolic equivalent minutes, physical fitness and musculoskeletal health

^c^Multivariable linear regression adjusted for covariates: age, sex, height, weekly metabolic equivalent minutes, physical fitness and cardiovascular health

^d^Indicates statistical significance < 0.05

^e^Indicates statistical significance < 0.01

^f^Indicates statistical significance < 0.001

In Table 5 we describe the associations between physical fitness, CVH and pass rates, using the predetermined cut-off times for each of the individual tasks. Firefighters who had a good abV̇O_2max_ had increased odds of passing the step-up (OR = 4.0), equipment carry (OR = 2.9), ladder raise and extension (OR = 2.8) and the rescue drag (OR = 1.9), respectively. Firefighters with good leg strength had increased odds of passing the forcible entry (OR = 11.6), equipment carry (OR = 1.9) and ladder raise and extension (OR = 1.9), respectively. Firefighters with good push-ups capacity had increased odds of passing the equipment carry (OR = 3.1), ladder raise and extension (OR = 3.1) and rescue drag (OR = 3.1). Firefighters with good sit-ups capacity had increased odds of passing step-up (OR = 3.6), equipment carry (OR = 2.2), ladder raise and extension (OR = 4.3) and rescue drag (OR = 2.4), respectively. For CVH, in the multivariable analyses, obese firefighters had decreased odds of passing the step-up task, those with a high BF% had decreased odds of passing the step-up (OR = 0.3), ladder raise and extension (OR = 0.4) and rescue drag (OR = 0.4) respectively. Physically inactive firefighters had decreased odds of passing the step-up (OR = 0.1), ladder raise and extension (OR = 0.5) and the rescue drag (OR = 0.3), respectively. Firefighters with an intermediate CVHI had increased odds of passing the equipment carry (OR = 2.1), ladder raise and extension (OR = 1.6) and the rescue drag (OR = 2.9), respectively, compared to firefighters with a poor CVHI. For MSH, upper body injuries (OR = 0.5) and low back injuries (OR = 0.3) decreased the odds of passing the rescue drag task. Firefighters that reported MSD and lower limb discomfort had decreased odds of passing the step-up (OR = 0.4 and 0.2), respectively. Low back discomfort decreased the odds of firefighters passing the rescue drag (OR = 0.4).

**Table 5:** Odds ratios describing the association between physical fitness, cardiovascular and musculoskeletal health and physical ability test task pass rates in firefighters

	Step-up		Charged hose drag and pull		Forcible entry		Equipment carry		Ladder raise and extension		Rescue drag	
	Model 1^a^	Model 2^b^	Model 1^a^	Model 2^b^	Model 1^a^	Model 2^b^	Model 1^a^	Model 2^b^	Model 1^a^	Model 2^b^	Model 1^a^	Model 2^b^
	OR (95% CI)	OR (95% CI)	OR (95% CI)	OR (95% CI)	OR (95% CI)	OR (95% CI)	OR (95% CI)	OR (95% CI)	OR (95% CI)	OR (95% CI)	OR (95% CI)	OR (95% CI)
Model: Demographics												
Age	2.1 (0.9, 4.8)	-	0.5 (0.2, 1.8)	-	0.8 (0.4, 1.7)	-	0.4 (0.3, 0.8)^d^	0.5 (0.2, 1.5)	0.54 (0.3, 0.9)^c^	0.4 (0.1, 0.9)^c^	1.6 (0.9, 2.9)	-
YoE (0–10 years)	0.9 (0.9, 0.9)^e^	0.8 (0.7, 0.9)^e^	-	-		-						
11–20 years	0.45 (0.14, 1.43)	-	0.1 (0.0, 0.6)^c^	0.1 (0.0, 1.4)	0.6 (0.3, 1.2)	-	0.6 (0.3, 1.2)	-	1.0 (0.6, 1.8)	-	0.6 (0.3, 1.0)	-
21–30 years	0.2 (0.1, 0.8)^c^	0.1 (0.0, 0.6)^c^	0.2 (0.0, 0.6)	-	1.4 (0.5, 3.9)	-	0.5 (0.3, 1.1)	-	0.8 (0.4,1.7)	-	0.8 (0.4, 1.7)	-
31 years and over	0.1 (0.0, 0.4)^e^	0.01 (0.0, 0.2)^d^	0.2 (0.0, 1.4)	-	0.4 (0.1, 0.9)	-	0.1 (0.1, 0.4)^e^	0.1^c^ (0.0, 0.9)	0.3 (0.2, 0.9)	-	0.1 (0.0, 0.3)^e^	0.1 (0.0, 0.6)^c^
Model: Physical fitness												
Ab. CRF	2.9 (1.3, 6.9)^c^	4.0 (1.2, 13.2)^c^	3.8 (1.2, 11.8)^c^	0.6 (0.1, 3.5)	2.9 (1.5, 5.7)^d^	1.4 (0.7, 3.1)	4.9 (2.8, 8.8)^e^	2.8 (1.4, 5.4)^d^	3.2 (1.9, 5.4)^e^	1.9 (1.1, 3.5)^c^	3.5 (2.0, 5.9)^e^	1.9 (1.1, 3.7)^c^
Rel. CRF	0.4 (0.2, 1.0)	-	1.1 (0.4, 2.8)	-	1.6 (0.9, 3.1)	-	0.9 (1.0, 1. 7)	-	1.3 (0.8, 2.2)	-	1.0 (0.1, 1.7)	-
Grip strength	1.4 (0.6, 3.2)	-	6.9 (1.5, 30.4)^c^	0.9 (0.1, 8.4)	3.6 (1.7, 7.6)^e^	2.2 (0.9, 5.1)	2.9 (1.6, 5.0)^e^	1.7 (0.9, 3.4)	3.9 (2.3, 6.6)^e^	2.6 (1.4, 4.6)^d^	2.5 (1.5, 4.2)^e^	1.3 (0.7, 2.5)
Leg strength	2.5 (0.9, 6.4)	-	11.9 (1.6, 90.5)^c^	1.4 (0.1, 21.5)	19.7 (4.7, 83.1)^e^	11.6 (2.7, 50.8)^d^	4.9 (2.6, 9.3)^e^	2.1 (1.0, 4.4)^c^	3.9 (2.3, 6.7)^e^	2.4 (1.4, 4.4)^d^	3.6 (2.0, 6.3)^e^	1.5 (0.8, 2.9)
Push-ups	4.1 (1.7, 10.1)^d^	2.4 (0.8, 6.7)	6.8 (1.9, 24.2)^d^	7.9 (0.8, 75.9)	2.1 (1.1, 4.0)^c^	1.8 (0.8, 4.0)	3.7 (2.1, 6.4)^e^	3.1 (1.5, 6.2)^d^	2.9 (1.7, 5.1)^e^	3.1 (1.6, 6.1)^e^	3.4 (2.0, 5.8)^e^	3.1 (1.5, 6.0)^d^
Sit-ups	5.2 (2.0, 13.1)^e^	3.6 (1.3, 10.1)^c^	4.7 (1.5, 14.7)^d^	4.9 (0.7, 32.6)	1.5 (.8, 2.9)	-	2.5 (1.5, 4.3)^e^	2.2 (1.1, 4.1)^c^	3.8 (2.2, 6.6)^e^	4.3 (2.2, 8.1)^e^	2.5 (1.5, 4.1)^e^	2.4 (1.3, 4.5)^d^
Flexibility	1.2 (0.5, 2.5)	-	1.2 (0.4, 2.9)	5.4 (0.9, 33.3)	1.2 (0.6, 2.2)	-	1.2 (0.7, 2.1)	-	1.5 (0.9, 2.5)	-	1.0 (0.6, 1.6)	-
Model: Cardiovascular health												
Obesity	0.2 (0.1, 0.4)^e^	0.2 (0.1, 0.6)^d^	0.2 (0.1, 0.4)^e^	0.2 (0.0, 1.2)	0.8 (0.4, 1.5)	-	0.5 (0.3, 0.9)^c^	0.9 (0.4, 1.8)	0.5 (0.3, 0.9)^c^	0.7 (0.3, 1.4)	0.5 (0.3, 0.9)^c^	0.8 (0.4, 1.5)
Central obesity	0.3 (0.1, 0.6)^d^	0.5 (0.2, 1.3)	0.3 (0.1, 0.8)^c^	0.6 (0.1, 3.3)	0.8 (0.4, 1.5)	-	0.5 (0.3, 0.8)^d^	0.7 (0.4, 1.4)	0.6 (0.4, 1.1)	-	0.5 (0.3, 0.8)^d^	0.8 (0.4, 1.5)
High BF%	0.2 (0.1, 0.4)^e^	0.3 (0.1, 0.8)^c^	0.1 (0.0, 0.3)^e^	0.3 (0.1, 1.6)	0.6 (0.3, 1.1)	-	0.5 (0.3, 0.8)^d^	0.9 (0.4, 1.8)	0.3 (0.1, 0.6)^e^	0.4 (0.2, 0.9)^c^	0.3 (0.2, 0.5)^e^	0.4 (0.2, 0.9)^c^
Hypertension	0.8 (0.4, 1.8)	-	0.5 (0.2, 1.3)	-	0.6 (0.3, 1.1)	-	0.9 (0.6, 1.6)		0.9 (0.6, 1.7)	-	1.1 (0.7, 1.9)	-
Diabetes	0.4 (0.1, 10.4)	-	0.0 (0.0)	-	0.4 (0.1, 3.4)	-	0.6 (0.2, 1.9)		0.6 (0.2, 2.2)	-	0.8 (0.2, 2.5)	-
Dyslipidaemia	0.6 (0.3, 1.3)	-	0.5 (0.2, 1.2)	-	1.1 (0.6, 2.1)	-	0.6 (0.3, 0.9)^c^	0.8 (0.4, 1.6)	0.8 (0.5, 1.4)	-	0.6 (0.4, 1.1)	-
High LDL-C	1.1 (0.5, 2.7)	-	0.9 (0.3, 2.7)	-	0.8 (0.4, 1.8)	-	0.7 (0.4, 1.3)		0.1 (0.5, 1.6)	-	0.9 (0.5, 1.7)	-
High HDL-C	1.1 (0.4, 3.1)	-	0.2 (0.0, 1.8)	-	1.4 (0.2, 10.9)	-	0.9 (0.5, 1.8)		0.9 (0.5, 1.8)	-	0.9 (0.5, 1.9)	-
Hypertriglyceridemia	1.3 (0.6, 3.1)	-	0.2 (0.0, 0.9)^c^	-	0.4 (0.2, 0.9)	-	0.8 (0.5, 1.3)		0.9 (0.6, 1.6)	-	0.8 (0.5, 1.4)	-
Physical inactivity	0.3 (0.1, 0.9)^c^	0.1 (0.1, 0.7)^c^	0.1 (0.0, 0.7)^c^	0.04 (0.0, 0.6)^c^	0.3 (0.2, 0.7)^d^	0.5 (0.1, 1.2)	0.4 (0.2, 0.7)^e^	0.6 (0.3, 1.2)	0.4 (0.2, 0.7)^e^	0.5 (0.2, 0.9)^c^	0.3 (0.2, 0.5)^e^	0.3 (0.1, 0.6)^d^
Cigarette smoking	2.1 (0.8, 5.5)		0.5 (0.2, 1.6)	-	0.8 (0.4, 1.6)	-	0.9 (0.6, 1.6)		0.7 (0.5, 1.5)	-	0.8 (0.5, 1.3)	0.5 (0.3, 0.9)^c^
CVHI (Poor)												
*Intermediate * *CVHI*	1.4 (0.6, 3.2)	-	2.2 (0.8, 5.9)	-	1.3 (0.7, 2.7)	-	2.5 (1.4, 4.4)^d^	2.1 (1.1, 4.1)^c^	1.9 (1.1, 3.5)^c^	1.6 (.9, 3.0)^c^	2.9 (1.6, 5.8)^d^	2.9 (1.6, 5.8)^d^
*Good CVHI*	2.2 (0.5, 10.4)	-	3.6 (0.4, 29.8)	-	0.7 (0.3, 1.8)	-	1.7 (7.0, 4.0)		1.1 (0.4, 2.6)		1.6 (0.7, 3.6)	
Model: Musculoskeletal health												
UBMSI	0.8 (0.3, 1,9)		0.6 (0.2, 1.9)	-	0.8 (0.3, 1.8)	-	0.6 (0.3, 1,2)	-	0.6 (0.3, 1.2)	-	0.5 (0.3, 0.9)^c^	0.5 (0.2, 0.9)^c^
LBMSI	0.9 (0.4, 2.2)		0.8 (0.3, 2.3)	-	1.5 (0.7, 3.5)	-	1.0 (0.5, 1.9)	-	0.8 (0.5, 1.5)	-	0.8 (0.4, 1.4)	-
LoBMSI	0.3 (0.1, 0.9)^c^	0.3 (0.1, 1.0)	0.6 (0.1, 2.9)	-	0.9 (0.3, 2.7)	-	0.4 (0.2, 1.0)	-	0.6 (0.2, 1.7)	-	0.3 (0.1, .9)^c^	0.3 (0.1, .8)^c^
Musculoskeletal discomfort	0.5 (0.2, 1.2)	0.4 (0.1, 0.9)^c^	0.7 (0.3, 1.9)	-	0.9 (0.6, 1.6)	-	1.3 (0.7, 2.1)	-	0.9 (0.6, 1.6)	-	0.7 (0.4, 1.2)	-
ULMSD	1.2 (0.5, 2.7)	-	0.7 (0.3, 1.8)	-	0.7 (0.4, 1.5)	-	1.1 (0.6, 2.2)	-	1.1 (0.7, 1.9)	-	0.6 (0.4, 1.0)	-
LLMSD	0.2 (0.1, 0.9)^c^	0.2 (0.0, 0.7)^c^	1.2 (0.4, 3.5)	-	0.8 (0.4, 1.8)	-	0.7 (0.4, 1.3)	-	0.7 (0.4, 1.2)	-	1.0 (0.6, 1.8)	-
LoBMSD	0.8 (0.3, 1.9)	-	0.3 (0.1, 0.8)^c^	0.4 (0.1, 1.9)^c^	0.5 (0.2, 1.0)	-	0.8 (0.4, 1.4)	-	0.8 (0.5, 1.6)	-	0.5 (0.3, 0.9)^c^	0.4 (0.2, 0.9)^c^

*ab. CRF* Absolute cardiorespiratory fitness, *rel. CRF* Relative cardiorespiratory fitness, *LDL-C* Low-density lipoprotein, *HDL-C* High-density lipoprotein, *BF%* Body fat percentage, *UBMSI* Upper body musculoskeletal injury, *LBMSI* Lower body musculoskeletal injury, *LoBMSI* Lower body musculoskeletal injury, *ULMSD* Upper limb musculoskeletal discomfort, *LBMSD* Lower body musculoskeletal discomfort, *LoBMSD* Lower back musculoskeletal discomfort

^a^Univariable models using logistic regression

^b^Multivariable logistic models adjusted for covariates: age, sex, height and weekly metabolic equivalent minutes

^c^Indicates statistical significance < 0.05

^d^Indicates statistical significance < 0.01

^e^Indicates statistical significance < 0.001

In Table 6 we further describe the associations between physical fitness, CVH and task pass rates, using the predetermined cut-off times for each of the individual tasks. Multivariable analysis included additional variables of CVH and physical fitness. For physical fitness, firefighters that had a good abV̇O_2max_ had an increased odds (OR = 4.3) of passing the step-up and ladder raise and extension (OR = 2.5) tasks. Firefighters with a good grip strength had an increase in odds of passing the forcible entry (OR = 2.4) and ladder raise and extension (OR = 2.5). Leg strength was associated with an increased odds (OR = 2.2) of passing the ladder raise and extension task. Firefighters with a good push-ups capacity was associated with an increased odds of passing the charged hose drag and pull (OR = 2.9) and the forcible entry (OR = 2.9) tasks. For CVH, obese firefighters had a decreased odds of passing the step-up (OR = 0.13), charged hose drag and pull (OR = 0.12), equipment carry (OR = 0.4), and rescue drag (OR = 0.3). Firefighters with an intermediate CVHI had an increased odds of passing the equipment carry (OR = 2.9), ladder raise and extension (OR = 1.9) and rescue drag (OR = 4.1) tasks. Firefighters with a good CVHI had an increased odds (OR = 3.4) of passing the rescue drag task. Firefighters with UBMSIs (OR = 0.4), LoBMSIs (OR = 0.2) and LoBMSD (OR = 0.4) had a decreased odds of passing the rescue drag task. Firefighters with MSD (OR = 0.3) and LLMSD (OR = 0.1) had a decreased odds of passing the step-up task.

**Table 6:** Odds ratios describing the association between physical fitness, cardiovascular and musculoskeletal health and physical ability test task pass rates in firefighters

	Step-up		Charged hose drag and pull			Forcible entry		Equipment carry		Ladder raise and extension		Rescue drag	
	Model 1^a^	Model 2^b^	Model 1^a^	Model 2^b^	Model 1^a^		Model 2^b^	Model 1^a^	Model 2^b^	Model 1^a^	Model 2^b^	Model 1^a^	Model 2^b^
	OR (95% CI)	OR (95% CI)	OR (95% CI)	OR (95% CI)	OR (95% CI)		OR (95% CI)	OR (95% CI)	OR (95% CI)	OR (95% CI)	OR (95% CI)	OR (95% CI)	OR (95% CI)
Model: Physical fitness													
Ab. CRF	4.3 (1.2, 15.7)^f^	-	2.3 (0.2, 24.5)	-	2.0 (0.8, 5.3)		-	2.1 (0.9, 5.0)	-	5.0 (2.1, 12.2)^f^	-	2.1 (0.9, 5.0)	-
Rel. CRF	2.4 (0.6, 9.0)	-	1.3 (0.2, 9.2)	-	2.0 (0.8, 5.3)		-	2.2 (0.9, 5.3)	-	5.0 (2.1, 12.2)^f^	-	2.1 (0.9, 5.0)	-
Grip strength	0.9 (0.9, 1.0)	-	1.3 (0.1, 14.5)	-	2.4 (1.0, 5.8)^d^		-	1.6 (0.8, 3.1)	-	2.5 (1.3, 4.6)^e^	-	1.2 (0.6, 2.4)	-
Leg strength	1.2 (0.4, 3.6)	-	1.4 (0.1, 19.9)	-	10.9 (2.5, 48.0)		-	1.9 (0.9, 4.0)	-	2.2 (1.2, 4.1)^d^	-	1.4 (0.7, 2.8)	-
Push-ups	1.3 (0.3, 4.9)	-	6.2 (0.4, 97.9)^e^	-	2.9 (1.1, 8.0)^d^		-	2.6 (1.1, 6.2)	-	1.3 (0.6, 2.9)	-	2.3 (0.9, 5.5)	-
Sit-ups	1.8 (0.5, 6.2)	-	2.7 (0.3, 24.2)	-	1.5 (0.6, 3.7)		-	1.7 (0.8, 3.9)	-	2.4 (1.1, 5.3)	-	1.9 (0.9, 4.3)	-
Flexibility	1.7 (0.6, 4.9)	-	0.3 (0.0, 2.3)	-	1.6 (0.9, 3.5)		-	0.9 (0.4, 1.8)	-	0.8 (0.4, 1.5)	-	0.6 (0.3, 1.2)	-
Model: Cardiovascular health													
Age	-	0.5 (0.2, 1.7)	-	0.5 (0.0, 4.5)	-		1.2 (0.5, 3.1)	-	0.4 (0.2, 0.9)^d^	-	0.9 (0.4, 1.0)	-	0.8 (0.4, 1.6)
Obesity	-	0.13 (0.0, 0.4)^f^	-	0.12 (0.0, 0.9)^d^	-		0.6 (0.2, 1.6)	-	0.4 (0.2, 0.9)^d^	-	0.2 (0.1, 0.5)	-	0.3 (0.1, 0.7)^e^
Central obesity	-	0.51 (0.2, 1.5)	-	0.1 (0.0, 0.5)^d^	-		0.8 (0.3, 2.2)	-	0.4 (0.2, 0.9)^d^	-	0.2 (0.1, 0.4)^f^	-	0.3 (0.1, 0.8)^d^
High BF%	-	0.3 (0.1, 0.7)^e^	-	0.3 (0.1, 1.5)	-		0.9 (0.4, 2.2)	-	0.6 (0.3, 1.4)	-	0.2 (0.1, 0.6)^e^	-	0.3 (0.1, 0.6)^e^
Hypertension	-	1.4 (0.5, 3.4)	-	0.4 (0.1, 1.9)	-		2.2 (0.9, 5.0)	-	1.3 (0.7, 2.7)	-	0.9 (0.5, 1.6)	-	1.3 (0.6, 2.6)
Diabetes	-	-	-	-	-		-	-	-	-	-	-	-
Dyslipidaemia	-	-	-	-	-		-	-	-	-	-	-	-
High LDL-C	-	-	-	-	-		-	-	-	-	-	-	-
High HDL-C	-	-	-	-	-		-	-	-	-	-	-	-
Hypertriglyceridemia	-	-	-	-	-		-	-	-	-	-	-	-
Physical inactivity	-	0.5 (0.2, 1.5)	-	0.03 (0.0, 0.5)^d^	-		0.8 (0.3, 2.1)	-	0.9 (0.4, 2.1)	-	0.7 (0.3, 1.3)	-	0.5 (0.3, 1.2)
Cigarette smoking	-	-	-	-	-		-	-	-	-	-	-	0.5 (0.3, 1.1)
CVHI (Poor)	-	-	-	-	-		-	-	-	-	-	-	-
*Intermediate CVHI*	-	-	-	-	-		-	-	2.9 (1.4, 6.2)^e^	-	1.9 (1.0, 3.9)^d^	-	4.1 (2.0, 8.4)^f^
*Good CVHI*	-	-	-	-	-		-	-	2.7 (0.8, 9.1)	-	1.9 (0.6, 5.7)	-	3.4 (1.0, 10.9)^d^
	Model 3^c^		Model 3^c^		Model 3^c^			Model 3^c^		Model 3^c^		Model 3^c^	
	OR (95% CI)		OR (95% CI)		OR (95% CI)			OR (95% CI)		OR (95% CI)		OR (95% CI)	
Model: Musculoskeletal health													
UBMSI	-	-	-	-	-		-	-	-	-	-	0.4 (0.2, 0.8)^d^	-
LBMSI	-	-	-	-	-		-	-	-	-	-	-	-
LoBMSI	0.4 (0.1, 1.3)	-	-	-	-		-	-	-	-	-	0.2 (0.1, 0.8)^d^	-
Musculoskeletal discomfort	0.3 (0.1, 0.9)^d^	-	-	-	-		-	-	-	-	-	-	-
ULMSD	-	-	-	-	-		-	-	-	-	-	-	-
LLMSD	0.1 (0.0, 0.7)^d^	-	-	-	-		-	-	-	-	-	-	-
LoBMSD	-	-	0.3 (0.0, 1.9)	-	-		-	-	-	-	-	0.4 (0.2, 0.9)^d^	-

*ab. CRF* Absolute cardiorespiratory fitness *rel. CRF* Relative cardiorespiratory fitness, *LDL-C* Low-density lipoprotein, *HDL-C* High-density lipoprotein, *BF%* Body fat percentage, *UBMSI* Upper body musculoskeletal injury, *LBMSI* Lower body musculoskeletal injury, *LoBMSI* Lower body musculoskeletal injury, *ULMSD* Upper limb musculoskeletal discomfort, *LBMSD* Lower body musculoskeletal discomfort, *LoBMSD* Lower back musculoskeletal discomfort

^a^Multivariable logistic regression adjusted for covariates: age, sex, height, weekly metabolic equivalents, cardiovascular health and musculoskeletal health

^b^Multivariable logistic regression adjusted for covariates: age, sex, height, weekly metabolic equivalent minutes, physical fitness and musculoskeletal health

^c^Multivariable logistic regression adjusted for covariates: age, sex, height, weekly metabolic equivalent minutes, physical fitness and cardiovascular health

^d^Indicates statistical significance < 0.05

^e^Indicates statistical significance < 0.01

^f^Indicates statistical significance < 0.001

In Table 7, the LASSO results for key indicators of physical fitness and CVH associated with occupational-specific task performance in firefighters are delineated. The results of the LASSO regression reported that abV̇O_2max_, grip strength, sit-ups, LBM, BF% and DBP were significant indicators for step-up completion times, explaining 26.6% of the variance. For the charged hose drag and pull, abV̇O_2max_, grip strength, leg strength, push-ups, sit-ups, LBM, age, BMI and weekly MET minutes were significant indicators and explained 55.6% of the variance in the task. For the forcible entry, abV̇O_2max_, grip strength, leg strength, sit-ups, LBM and weekly MET minutes remained significant indicators of completion time in the task, explaining 26.2% of the variance. AbV̇O_2max_, grip strength, leg strength, push-ups, sit-ups, sit-and-reach, LBM, age, BMI, BF%, HDL-C and weekly MET minutes were significant indicators of performance on the equipment carry and explained 45.3% of the variance in the task. For the ladder raise and extension abV̇O_2max_, grip strength, leg strength, sit-ups, LBM BF% and Weekly MET minutes were significant indicators of task completion times and explained 42.1% of the variance in the task. For the rescue drag, abV̇O_2max_, grip strength, leg strength, push-ups, sit-ups, LBM, age and weekly MET minutes explain 47.2% of the variance in the task performance.

**Table 7:** LASSO-derived multivariable linear regression coefficients to discern key physical fitness and CVH parameters most associated with task performance in firefighters

Model summary	Step-up	CHDP	FE	EC	LRE	RD
Prediction	0.791	0.463	0.757	0.574	0.629	0.561
Estimate	0.832	0.507	0.814	0.614	0.709	0.618
R^2^	0.266	0.556	0.292	0.453	0.421	0.472
Variables						
abV̇O_2max_ (L•min)	-0.007	-0.092	-0.106	-0.191	-0.108	-0.065
relV̇O_2max_ (mL•kg•min)	-	-	-	-	-	-
Grip strength (kg)	-0.072	-0.169	-0.064	-0.138	-0.201	-0.168
Leg strength (kg)	-	-0.134	-0.105	-0.036	-0.053	-0.105
Push-ups (rpm)	-	-0.117	-	-0.136	-	-0.151
Sit-ups (rpm)	-0.177	-0.086	-0.037	-0.059	-0.137	-0.068
Sit-and-reach (cm)	-	-	-	-0.003	-	-
Lean body Mass (kg)	-0.066	-0.278	-0.121	-0.112	-0.134	-0.185
Age (years)	-	0.093	-	0.043	-	0.022
Body mass index (kg•m^−2^)	-	0.073	-	0.001	-	-
Waist circumference (cm)	-	-	-	-	-	-
Body fat percentage (%)	0.092	-	-	0.091	0.077	-
Systolic blood pressure (mmHg)	-	-	-	-	-	-
Diastolic blood pressure (mmHg)	0.018	-	-	-	-	-
Non-fasting blood glucose (mmol•L^−1^)	-	-	-	-	-	-
Total cholesterol (mmol•L^−1^)	-	-	-	-	-	-
Low-density lipoprotein cholesterol (mmol•L^−1^)	-	-	-	-	-	-
High-density lipoprotein cholesterol (mmol•L^−1^)	-	-	-	-0.001	-	-
Triglycerides (mmol•L^−1^)	-	-	-	-	-	-
Weekly MET minutes (MET•min)	-	-0.009	-0.028	-0.112	-0.051	-0.068
Framingham risk score (%)	-	-	-	-	-	-

*R*^*2*^ R squared, *CHDP* Charged hose drag and pull, *FE* Forcible entry, *EC* Equipment carry, *LRF* Ladder raise and extension, *RD* Rescue drag, *kg•m*^*−2*^ Kilogram per meter squared, *cm* Centimetre, *%* Percentage, *mm Hg* Millimetres of mercury, *mmol•L*^*−1*^ Millimole per litre, *MET* Metabolic equivalents, *BMI* Body mass index, *WC* Waist circumference, *SBP* Systolic blood pressure, *DBP* Diastolic blood pressure, *NFBG* Non-fasting blood glucose, *TC* Total cholesterol, *LDL-C* Low-density lipoprotein, *HDL-C* High-density lipoprotein, *rpm* Repetitions per minute

## Discussion

The results of the study indicated that firefighters with higher levels of absolute cardiorespiratory fitness, muscle strength and endurance and favourable body composition, performed all occupational-specific tasks significantly faster and were more likely to pass each task. This is consistent with previous studies where higher levels of physical fitness was related to better occupational-specific task performance in firefighters [3, 5, 6, 52]. In addition, the results indicated that firefighters aged 45 years and older who had a BMI over 30 kg•m^−2^ and those that had higher blood pressure, worse lipid profile and a low HRV were the poorest performers on all the individual occupational-specific tasks. These results corroborate previous research where older and obese firefighters had poorer performance on most occupational tasks [3, 5, 6]. Moreover, higher levels of blood pressure and worse lipid profile have been shown to be associated with lower levels of physical fitness [53–55], providing a potential explanation for poorer performance on the individual tasks in this group. In the present study, firefighters that reported sustaining an MSI performed the rescue drag task significantly slower and those that reported more MSD performed the step-up, charged hose drag and pull and the rescue drag task significantly slower. This is consistent with previous studies where MSH was related to more physical and work functioning restrictions [26, 27, 30].

In the current study, an increase in absolute cardiorespiratory fitness was associated with faster completion times for all occupational-specific tasks and a key indicator in the performance of all occupational-specific tasks, which remained significant after adjustment for CVH and MSH. However, relative cardiorespiratory fitness was related to faster completion times for the step-up task, only. Schonfeld et al. [56] reported that relV̇O_2max_ was inversely related to a stair climb (*r* = -0.627), chopping task (*r* = -0.324) and the victim rescue (*r* = -0.447) tasks in firefighters. Similarly, Chizewski et al. [3] found estimated relV̇O_2max_ was inversely related to the self-contained breathing apparatus (SCBA) crawl (*r* = -0.530), victim rescue (*r* = -0.342), hose advance (*r* = -0.266) and the equipment carry (*r* = -0.361) tasks. Studies have suggested that occupational tasks that require more time to complete, that are also more strenuous, require higher levels of cardiorespiratory fitness to perform them adequately [3, 5, 6, 52]. Moreover, we found that after adjustment for age, sex, height and weekly MET minutes, CVH and MSH, absolute cardiorespiratory fitness remained significantly related to all tasks. Furthermore, absolute cardiorespiratory fitness, rather than relative cardiorespiratory fitness, contributed more significantly toward overall occupational-specific task performance. A study by Perroni et al. [57] also found that absolute cardiorespiratory fitness was more correlated to performance of the Queens College Step Field test compared to relative cardiorespiratory fitness (*r* = 0.76 vs *r* = 0.54) while performing the test wearing full PPE. The authors noted that using absolute oxygen may be a useful tool when evaluating cardiovascular strain in firefighters while firefighters are in PPE [57]. It is possible that absolute cardiorespiratory fitness may be a valuable measure while firefighters are wearing full PPE, as higher levels of relative oxygen consumption may not necessarily relate to better performance if firefighters lack the necessary muscle mass and strength needed to overcome the additional weight [57, 58]. Although being leaner may be more favourable in many cases, a higher overall LBM reflecting a greater muscular mass/strength and a greater ability to utilize oxygen (absolute oxygen utilisation) [59], may explain more favourable performances on each of the occupational-specific tasks. This would suggest that firefighters with a higher LBM, regardless of body weight, and a higher absolute V̇O_2max,_ would perform significantly better, likely due to greater oxygen uptake and additional muscular strength to overcome the weight of their PPE [3, 33, 39, 60–62]. This is supported by the results of the present study, where we found that firefighters that had a higher LBM had significantly shorter completion times on all occupational-specific tasks. This was further corroborated by studies by Williford et al. [5], Davis et al. [34] and Henderson et al. [58] reported that higher LBM was negatively associated with individual task performance. It is likely firefighters with a higher LBM are taller and heavier, with more muscle mass, which has all been shown to be related to better performance on all tasks [5, 17, 39]. Von Heimburg et al. [62] found that peak V̇O_2_ could accurately predict occupational performance and more so when expressed as absolute cardiorespiratory fitness rather than relative cardiorespiratory fitness. Possibly peak V̇O_2_ (absolute) may be important for faster occupational performance, and for slower, less fit firefighters, accumulated V̇O_2_ or the ability to sustain a minimum V̇O2 may be crucial in completing their occupational-specific tasks.

We found that higher muscular strength and muscular endurance was associated with shorter completion times for all individual occupational-specific tasks. In addition, this remained significant when adjusted for CVH and MSH in the multivariable Models. Michaelides et al. [61] reported that push-ups stamina and muscular strength was related to better performance on individual tasks. Williford et al. [5] corroborated these findings, reporting that grip strength was negatively related to the forcible entry task (*r* = -0.53), equipment hoist (*r* = -0.55), hose advance (*r* = -0.41), victim rescue (*r* = -0.59) and stair climb tasks (*r* = -0.39). This was further supported by Skinner et al. [18] who reported that higher strength levels in the bench press (*r* = -0.471) and higher endurance capacity in the push-ups (*r* = -0.385) were negatively related to the hose drag task. Von Heimburg et al. [62] noted that there was a minimum standard of muscular strength and endurance are required to perform the occupational tasks acceptably and muscular strength exceeding this point had progressively less impact on the performance of each task. Moreover, overweight and obese firefighters with higher strength levels did not perform better than fighters who weighed less that had sufficient strength to overcome the task [62], which had been a finding that was reported by Phillips et al. [17]. In the present study, we found that higher sit-and-reach scores were associated to lower completion times on the equipment carry task. A systematic review [52] reported that there was a significant effect for flexibility on the stair climb task in firefighters. However, results for the relationship between flexibility and task performance are inconsistent in the literature [3, 18, 52, 60].

In the current study we found that as age (and hence years of experience) increased, the completion times for each of the occupational-specific performance tasks increased. However, when adjusted for physical fitness and MSH, significances were removed. Previous studies have found similar results, indicating aging was negatively related to occupational-specific task performance in firefighters [3, 5, 18]. This may be due to the natural age-related decrease in cardiorespiratory fitness, muscular strength and endurance, negatively affecting occupational performance in firefighters [63–66]. Researchers have argued that older and more experienced firefighters have learned superior techniques that could, at least partially, counteract the age-related decrease in cardiorespiratory fitness [39].

We found that an increase in BF% and BMI were associated with significantly slower completion times for all occupational-specific tasks in firefighters, which remained significant after adjustment for physical fitness and MSH. Previous study reported similar results where an increase in BF% was related to slower completion times for each task [3, 5], particularly the stair climb task, where firefighters are required to traverse stairs, carrying their bodyweight in addition to a high-rise pack [56, 67]. An increase in body fat represents non-functional mass that increases the effort firefighters are required to exert to successfully complete each task, which, subsequently, increases the time taken to complete each task [52, 60, 61]. It is also plausible that obese firefighters ambulate more slowly and less efficiently [68], extending the time to complete each task that requires continual movement, such as the hose drag, equipment carry or victim drag, while also requiring additional time moving from task to task. In addition, it is likely that obese firefighters’ fatigue quicker, consequently reducing their overall occupational performance [5, 67, 69]. The findings of the present study indicated that a higher blood pressure was associated with an increase in the step-up, the charged hose drag and pull, and the equipment carry completion times. Similarly, Davis et al. [34] reported that diastolic blood pressure was positively related to occupational task performance (*r* = 0.233) in firefighters. In the present study, the step-up, charged hose drag and pull and the equipment carry tasks involved strong isometric and isotonic contractions, which leads to an exaggerated blood pressure response [20, 70].

We found that an increase in HRV, SDNN and RMSSD was associated with faster completion times for all occupational-specific tasks, and LF range was associated with better performance on all tasks, except the forcible entry. After adjustment for physical fitness, CVH and MSH, SDNN and RMSSD remained significantly associated to certain occupational-specific tasks. A study by Lesniak et al. [71] reported that SDNN was negatively related to the hose drag (*r* = -0.745), ladder raise (*r* = -0.738) and rescue (*r* = -0.738) tasks and LF/HF ratio was negatively related to the forcible entry task (*r* = -0.718). Previous studies have also found that firefighters that had higher HRV was related to higher physical performance [72, 73], sleepiness and higher levels of fatigue [74], and cardiovascular health [75]. Theoretically, Firefighters with higher HRV indices would be fitter, and healthier, consequently performing better on of all the occupational-specific tasks. The LF range has been reported to be associated with the physical fitness levels, the stress state and baroreceptor functioning in individuals [76]. This suggests that firefighters that are in lower stress states are fitter and may perform their duties more efficiently than those that are in a more stressed state, which has been a proposed theory explaining the reasons for performance decrements in firefighters [76–78]. This becomes particularly evident as firefighters age and become more stressed, as a result of being in the profession for a longer period [79, 80].

We found that taller and heavier firefighters performed significantly better than their lighter and shorter counterparts. This was consistent with a study conducted by Phillips et al. [17] that reported heavier and, subsequently, taller, firefighters performed favourably on all simulation tasks, except the ladder climb test. Similarly, Williford et al. [5] reported that height and weight were significantly related to all occupational performance task completion times. Taller firefighters, inherently, would have a higher LBM, consequently, a higher overall muscle mass and V̇O_2max_ [17, 18, 81]. Von Heimburg et al. [39] separated participants into fast and slow performers, and found that those who performed a rescue operation fastest were taller (9 cm) and heavier (10 kg more) than those who performed the task more slowly.

Firefighters that reported MSIs had slower completion times for the step-up and rescue drag tasks and those with MSD, particularly in the lower back region, had slower completion times for the step-up, charged hose drag and pull and rescue drag tasks, which remained significant after the addition of physical fitness and CVH as covariates. McDermid et al. [27] reported that MSD was not significantly related to the completion times of the stair climb or hose drag tasks. However, firefighters with severe discomfort took 10 s longer to perform the stair climb compared to those without discomfort. Similarly, Nazari et al. [82] reported that spine pain was related to firefighters reporting the most physical and work limitations. In addition, the current study showed that firefighters who experienced more overall MSD and, those specifically experiencing MSD in the shoulder, upper back, wrist and hand regions took significantly longer to complete the forcible entry task. Since the forcible entry task requires firefighters to swing a sledgehammer with maximal force [3, 5], it is unsurprising that firefighters with MSD in the shoulder, upper back and wrist and hand regions would have the most physical limitations leading to worse performance. Azmi and Masuri [30] reported that MSD in the upper back, lower back, left wrist and left thigh contributed to 50% of the limitation to functional status in firefighters. Limitations, caused by previous injury or current discomfort, may contribute toward firefighters guarding the injured or discomforted area [26, 27]. Moreover, pain or previous injury may contribute toward reduced force production contributing toward worse performance on each task, particularly those requiring weight bearing, placing stain on the lower limbs and low back, such as the step-up, charged hose drag and pull and the rescue drag, as seen in the present study [83].

The results of the LASSO analysis indicated that firefighters with higher cardiorespiratory fitness, muscle endurance capacity, who are stronger, more physically active and had a lower BF% and higher LBM had the shortest completion time on the step-up, charged hose drag and pull, forcible entry, equipment carry, ladder raise and extension and the rescue drag tasks. Previous studies are consistent with these findings, and have shown that stronger, fitter and leaner firefighters performed the stair climb, hose drag and pull, forcible entry, equipment carry, ladder raise and rescue drag tasks significantly quicker than weaker, overweight/obese and less fit firefighters [3, 6, 18, 34, 61].

### Strengths and limitations

This was the first study to investigate the association between physical fitness, cardiovascular and musculoskeletal health in relation to occupational-specific task performance through a physical ability test performed by firefighters in the CoCTFRS, adding novel findings, particularly in a South African context. The measures for physical fitness, cardiovascular health, and occupational-specific task performance were objectively measured by trained researchers, using standardized and validated instruments [35]. There are, however, several limitations to the present study. The first limitation is the cross-sectional study design which precludes the inference of causal relationships. A second limitation was that female firefighters were underrepresented, limiting the generalizability to the female firefighter population. Cardiorespiratory fitness was measured using a non-exercise estimation, not using lab or field testing. Lastly, the multiple comparisons on the relatively small sample size may have increased the possibility of spurious findings.

## Conclusion

The present study showed that multiple parameters of physical fitness, cardiovascular health, and musculoskeletal health were related to better occupational-specific task performance in firefighters. Fitter, more active, stronger, and leaner firefighters who had a more favourable cardiovascular health profile, and without musculoskeletal health concerns were the best performers on each occupational-specific task. Moreover, firefighters with higher HRV showed faster performance in all occupational-specific tasks, providing novel findings on the relationship between cardiovascular autonomic functioning and work performance in firefighters. The use of HRV may provide a useful, and relatively cost effective, criterion in assessing the physical fitness, cardiovascular health, and occupational performance of firefighters. Municipal fire departments may use the study's findings to emphasize the necessity for physical fitness and cardiovascular health standards to improve firefighters’ occupational performance, as well as to protect the cardiovascular health and musculoskeletal health of firefighters, and increase the longevity of their careers. Fire departments can enhance the services they offer, lower the risk of civilian casualties, and prevent damage to vital infrastructure by instituting regular physical exercise programs and enforcing a basic fitness standard for all firefighters.

### Supplementary Information

**Additional file 1:** **Supplementary Table:** Odds ratios describing the interrelationship between physical fitness, cardiovascular and musculoskeletal health and physical ability test task pass rates in firefighters.

## Data Availability

The datasets generated and/or analysed during the current study are not publicly available due to the POPIA act in South Africa, limiting the distribution of information on research participants; but are available from the corresponding author on reasonable request.
