# Supplementary material for: Evaluation of the relationship between occupational-specific task performance and measures of physical fitness, cardiovascular and musculoskeletal health in firefighters
Source: BMC Public Health. 2024 Jan 2;24:20. doi: 10.1186/s12889-023-17487-6 (PMC10763081; doi:10.1186/s12889-023-17487-6)
Supplement: Supplementary file 1 — Additional file 1: Supplementary Table: Odds ratios describing the interrelationship between physical fitness, cardiovascular and musculoskeletal health and physical ability test task pass rates in firefighters. [file 12889_2023_17487_MOESM1_ESM.docx]

| Supplementary Table: Odds ratios describing the interrelationship between physical fitness, cardiovascular and musculoskeletal health and physical ability test task pass rates in firefighters. | | | | | | | | | | | | | | | | | | | | | |  |
| --- | --- | --- | --- | --- | --- | --- | --- | --- | --- | --- | --- | --- | --- | --- | --- | --- | --- | --- | --- | --- | --- | --- |
|  | Step-up | | | Charged hose drag and pull | | | Forcible entry | | | | Equipment carry | | | Ladder raise and extension | | | | Rescue drag | | | |  |
|  | Model 1 ^a^ | Model 2 ^b^ | | Model 1 ^a^ | | Model 2 ^b^ | Model 1 ^a^ | | Model 2 ^b^ | Model 1 ^a^ | | Model 2 ^b^ | | Model 1 ^a^ | | Model 2 ^b^ | | Model 1 ^a^ | | Model 2 ^b^ | |  |
|  | OR  (95% CI) | OR  (95% CI) | | OR  (95% CI) | | OR  (95% CI) | OR  (95% CI) | | OR  (95% CI) | OR  (95% CI) | | OR  (95% CI) | | OR  (95% CI) | | OR  (95% CI) | | OR  (95% CI) | | OR  (95% CI) | |  |
| Model: Physical fitness |  |  | |  | |  |  | |  |  | |  | |  | |  | |  | |  | |  |
| Ab. CRF | 2.4 (1.0, 5.8) * |  | | 4.1 (1.3, 12.9) * | |  | 3.0 (1.5, 6.1) ** | |  | 6.0 (3.2, 11.2) † | |  | | 3.7 (2.1, 6.4) † | |  | | 4.5 (2.5, 8.1) † | |  | |  |
| Rel. CRF | 1.4 (0.5, 4.4) | - | | 1.6 (0.4, 5.9) | | - | 2.2 (0.9, 5.4) | | - | 2.2 (1.1, 4.7) * | | - | | 4.5 (2.2, 9.4) † | | - | | 1.9 (0.9, 3.9) | | - | |  |
| Grip strength | 1.3 (0.54, 2.0) |  | | 6.8 (1.5, 30.3) * | |  | 3.9 (1.8, 8.4) † | |  | 2.7 (1.5, 4.8) † | |  | | 3.9 (2.2, 6.8) † | |  | | 2.4 (1.4, 4.1) ** | |  | |  |
| Leg strength | 1.9 (0.7, 4.9) |  | | 10.8 (1.4, 83.4) * | |  | 19.1 (4.5, 81.6) † | |  | 4.6 (2.4, 9.0) † | |  | | 3.4 (1.9, 5.9) † | |  | | 3.2 (1.8, 5.8) † | |  | |  |
| Push-ups | 1.9 (0.6, 6.1) |  | | 11.9 (2.6, 56.0) ** | |  | 4.6 (1.8, 11.3) ** | |  | 3.9 (1.9, 8.5) † | |  | | 2.2 (1.1, 4.6) * | |  | | 3.4 (1.6, 7.2) ** | |  | |  |
| Sit-ups | 2.9 (0.9, 8.9) |  | | 6.1 (1.6, 23.6) ** | |  | 2.1 (0.9, 4.7) | |  | 2.2 (1.1, 4.3) * | |  | | 3.2 (1.6, 6.5) † | |  | | 2.1 (1.1, 4.2) * | |  | |  |
| Flexibility | 0.4 (0.2, 1.2) |  | | 0.8 (0.3, 2.3) | |  | 1.3 (0.6, 2.7) | |  | 0.8 (0.4, 1.5) | |  | | 0.9 (0.5, 1.6) | |  | | 0.6 (0.3, 1.0) | |  | |  |
|  |  |  | |  | |  |  | |  |  | |  | |  | |  | |  | |  | |  |
|  | | |  | |  |  | |  | | |  | |  | |  | |  | |  | |  | |
| Age |  | 0.8 (0.3, 1.9) | |  | | 1.6 (0.3, 7.9) |  | | 1.2 (0.5, 2.9) |  | | 0.4 (0.2, 0.8) ** | |  | | 0.8 (0.4, 1.6) | |  | | 0.8 (0.4, 1.5) | |  |
| Obesity |  | 0.3 (0.1, 1.1) | |  | | 0.1 (0.0, 0.9) * |  | | 0.3 (0.1, 1.3) |  | | 0.4 (0.1, 1.1) | |  | | 0.3 (0.1, 0.9) * | |  | | 0.5 (0.2, 1.5) | |  |
| Central obesity |  | 1.2 (0.3, 4.3) | |  | | 0.1 (0.0, 0.7) * |  | | 0.7 (0.2, 2.2) |  | | 0.3 (0.1, 0.9) * | |  | | 0.3 (0.1, 0.8) * | |  | | 0.5 (0.2, 1.3) | |  |
| High BF% |  | 0.8 (0.2, 2.9) | |  | | 0.8 (0.1, 6.1) |  | | 0.9 (0.3, 2.9) |  | | 1.3 (0.5, 3.5) | |  | | 0.4 (0.1, 1.3) | |  | | 0.5 (0.2, 1.4) | |  |
| Hypertension |  | 1.3 (0.5, 3.3) | |  | | 0.3 (0.1, 1.1) |  | | 1.9 (0.8, 3.8) |  | | 1.0 (0.5, 1.9) | |  | | 1.0 (0.6, 1.9) | |  | | 1.3 (0.7, 2.5) | |  |
| Diabetes |  | 0.6 (0.1, 2.9) | |  | | - |  | | 0.9 (0.2, 4.9) |  | | 1.6 (0.2, 13.9) | |  | | 0.9 (0.2, 4.9) | |  | | 1.3 (0.3, 5.1) | |  |
| Dyslipidaemia |  | 0.6 (0.2, 1.5) | |  | | 0.1 (0.0, 0.6) * |  | | 0.8 (0.4, 1.7) |  | | 0.5 (0.2, 0.9) * | |  | | 0.9 (0.4, 1.7) | |  | | 0.6 (0.3, 1.2) | |  |
| High LDL-C |  | 1.3 (0.5, 3.5) | |  | | 0.5 (0.1, 2.6) |  | | 1.2 (0.5, 2.9) |  | | 0.6 (0.3, 1.3) | |  | | 0.8 (0.4, 1.7) | |  | | 0.9 (0.5, 2.0) | |  |
| High HDL-C |  | 0.9 (0.3, 3.0) | |  | | 0.9 (0.1, 9.9) |  | | 0.6 (0.2, 1.6) |  | | 0.7 (0.3, 1.5) | |  | | 0.8 (0.4, 1.7) | |  | | 0.7 (0.3, 1.5) | |  |
| Hypertriglyceridemia |  | 1.9 (0.7, 5.1) | |  | | 2.6 (0.4, 15.9) |  | | 1.8 (0.8, 4.1) |  | | 1.3 (0.7, 2.6) | |  | | 1.3 90.6, 2.5) | |  | | 0.9 (0.5, 1.8) | |  |
| Physical inactivity |  | 1.1 (0.4, 2.9) | |  | | 0.1 (0.0, 1.3) |  | | 0.5 (0.2, 1.2) |  | | 0.7 (0.3, 1.3) | |  | | 0.8 (0.4, 1.5) | |  | | 0.5 (0.3, 1.0) | |  |
| Cigarette smoking |  | 1.7 (0.6, 4.7) | |  | | 0.7 (0.1, 3.3) |  | | 0.9 (0.4, 1.9) |  | | 0.9 (0.5, 1.6) | |  | | 1.0 (0.5, 1.9) | |  | | 0.7 (0.4, 1.3) | |  |
| CVHI (Poor) |  |  | |  | |  |  | |  |  | |  | |  | |  | |  | |  | |  |
| *Intermediate CVHI* |  | 6.5 (1.1, 36.9) * | |  | | 6.5 (1.1, 36.9) * |  | | 1.9 (0.8, 4.5) |  | | 3.3 (1.6, 6.7) ** | |  | | 1.5 (0.7, 3.1) | |  | | 0.5 (0.1, 1.6) | |  |
| *Good CVHI* |  | 27.2 (1.3, 550.8) * | |  | | 27.2 (1.3, 550.8) * |  | | 1.6 (0.4, 5.7) |  | | 3.9 (1.2, 12.9) * | |  | | 1.0 (0.3, 3.5) | |  | | 1.7 (0.5, 4.9) | |  |
|  |  |  | |  | |  |  | |  |  | |  | |  | |  | |  | |  | |  |
|  | Model 3 ^c^ | | | Model 3 ^c^ | | | Model 3 ^c^ | | | Model 3 ^c^ | | | | Model 3 ^c^ | | | | Model 3 ^c^ | | | |  |
|  | OR (95% CI) | | | OR (95% CI) | | | OR (95% CI) | | | OR (95% CI) | | | | OR (95% CI) | | | | OR (95% CI) | | | |  |
| Model: Musculoskeletal health |  |  | |  | |  |  | |  |  | |  | |  | |  | |  | |  | |  |
| UBMSI | 0.9 (0.3, 2.5) |  | | 0.8 (0.2, 4.8) | |  | 0.9 (0.4, 2.3) | |  | 0.6 (0.3, 1.3) | |  | | 0.5 (0.2, 1.2) | |  | | 0.4 (0.2, 0.8) * | |  | |  |
| LBMSI | 1.3 (0.5, 3.4) |  | | 2.3 (0.4, 11.6) | |  | 2.1 (0.8, 5.5) | |  | 1.3 (0.6, 2.7) | |  | | 1.1 (0.5, 2.2) | |  | | 0.9 (0.4, 1.9) | |  | |  |
| LoBMSI | 0.3 (0.1, 1.0) |  | | 0.9 (0.1, 13.3) | |  | 0.9 (0.2, 3.7) | |  | 0.4 (0.1, 1.2) | |  | | 0.6 (0.2, 2.1) | |  | | 0.3 (0.1, 0.9) * | |  | |  |
| Musculoskeletal discomfort | 2.5 (0.9, 6.6) |  | | 1.1 (0.3, 4.4) | |  | 0.9 (0.5, 2.1) | |  | 1.4 (0.7, 2.6) | |  | | 0.9 (0.5, 1.6) | |  | | 0.6 (0.3, 1.1) | |  | |  |
| ULMSD | 1.3 (0.5, 3.3) |  | | 0.6 (0.1, 2.5) | |  | 0.8 (0.4, 1.8) | |  | 1.2 (0.6, 2.2) | |  | | 1.2 (0.6, 2.2) | |  | | 0.4 (0.2, 0.8) * | |  | |  |
| LLMSD | 0.2 (0.0, 0.9) * |  | | 0.9 (0.2, 4.4) | |  | 0.7 (0.3, 1.7) | |  | 0.6 (0.2, 1.4) | |  | | 0.7 (0.3, 1.4) | |  | | 0.8 (0.4, 1.8) | |  | |  |
| LoBMSD | 0.9 (0.3, 2.5) |  | | 0.3 (0.1, 1.4) | |  | 0.5 (0.2, 1.2) | |  | 0.9 (0.4, 1.9) | |  | | 0.6 (0.3, 1.3) | |  | | 0.5 (0.2, 0.9) * | |  | |  |
|  |  |  | |  | |  |  | |  |  | |  | |  | |  | |  | |  | |  |
| **Note:** * − Indicates statistical significance <0.05; ** − Indicates statistical significance <0.01; † − indicates statistical significance <0.001.  a – multivariable logistic regression adjusted for covariates: cardiovascular health and musculoskeletal health; b – Multivariable logistic regression adjusted for covariates: physical fitness and musculoskeletal health; c – Multivariable logistic regression adjusted for covariates: physical fitness and cardiovascular health; ab. CRF – absolute cardiorespiratory fitness; rel. CRF – relative cardiorespiratory fitness; LDL-C – low-density lipoprotein; HDL-C – high-density lipoprotein; BF% − body fat percentage; UBMSI − upper body musculoskeletal injury; LBMSI − lower body musculoskeletal injury; LoBMSI − lower body musculoskeletal injury; ULMSD − upper limb musculoskeletal discomfort; LBMSD − lower body musculoskeletal discomfort; LoBMSD − lower back musculoskeletal discomfort. | | | | | | | | | | | | | | | | | | | | | |  |
